# Mutations targeting the coagulation pathway are enriched in brain metastases

**DOI:** 10.1038/s41598-017-06811-x

**Published:** 2017-07-26

**Authors:** Cristina Richichi, Lorenzo Fornasari, Giorgio E. M. Melloni, Paola Brescia, Monica Patanè, Massimiliano Del Bene, Dana A. M. Mustafa, Johan M. Kros, Bianca Pollo, Giancarlo Pruneri, Angela Sciandivasci, Elisabetta Munzone, Francesco DiMeco, Pier Giuseppe Pelicci, Laura Riva, Giuliana Pelicci

**Affiliations:** 1 Department of Experimental Oncology, European Institute of Oncology, 20139 Milano, Italy; 2 Center for Genomic Science of IIT@SEMM, Fondazione Istituto Italiano di Tecnologia, 20139 Milano, Italy; 3 Department of Neuropathology, IRCCS Foundation Neurological Institute “C. Besta”, 20133 Milano, Italy; 4 Department of Neurosurgery, IRCCS Foundation Neurological Institute “C. Besta”, 20133 Milano, Italy; 5 Department of Pathology, Erasmus University Medical Center, 3015 Rotterdam, The Netherlands; 6 Division of Pathology, European Institute of Oncology, 20139 Milano, Italy; 7 University of Milan, Breast Cancer Program, 20122 Milano, Italy; 8 Division of Medical Senology, European Institute of Oncology, 20141 Milano, Italy; 9 Department of Neurosurgery, Johns Hopkins University, Baltimore, MD 21218 USA; 10 Department of Oncology and Hemato-oncology, University of Milano, 20122 Milan, Italy; 11 Department of Translational Medicine, Piemonte Orientale University “Amedeo Avogadro”, 28100 Novara, Italy

## Abstract

Brain metastases (BMs) are the most common malignancy of the central nervous system. Recently it has been demonstrated that plasminogen activator inhibitor serpins promote brain metastatic colonization, suggesting that mutations in serpins or other members of the coagulation cascade can provide critical advantages during BM formation. We performed whole-exome sequencing on matched samples of breast cancer and BMs and found mutations in the coagulation pathway genes in 5 out of 10 BM samples. We then investigated the mutational status of 33 genes belonging to the coagulation cascade in a panel of 29 BMs and we identified 56 Single Nucleotide Variants (SNVs). The frequency of gene mutations of the pathway was significantly higher in BMs than in primary tumours, and *SERPINI1* was the most frequently mutated gene in BMs. These findings provide direction in the development of new strategies for the treatment of BMs.

## Introduction

Brain metastases (BMs) are the most common malignancy of the central nervous system, and their incidence is dramatically increasing due to the improvements in overall survival of primary tumour patients and failure of systemic chemotherapy to reach the central nervous system^1^. Mainly, BMs derive from primary tumours originating in the breast, lung, skin, gastrointestinal tract and kidney^2, 3^.

Breast cancer (BC) is a heterogeneous disease that remains the second leading cause of death among women worldwide^4^: up to 16% of patients with metastatic BC develop symptomatic BMs during the course of their disease, and another 10% of patients have asymptomatic brain involvement in post-mortem autopsies^5, 6^. Because of improvements in the treatment of patients with metastatic breast cancer, the development of brain metastases has become a major impediment to improved life expectancy and quality of life for many breast cancer patients. The median survival time is very short, ranging from 2 to 25 months despite treatment^7–10^. In addition, the intra-tumour and inter-tumour heterogeneity of BC influences the clinical course of the disease and the response to treatment.

Although some gene expression signatures have been previously associated with metastasis to the brain^11, 12^, the driver mutations that lead to the formation of BM are still largely unknown. Although the frequency of these mutations is fairly low, the metastatic spread is sustained by gene mutations conferring selective growth advantage to target cells^13–15^. Large-scale genome sequencing of human tumours found little evidence for specific metastasis-associated mutations other than mutations in classic initiator oncogenes (driver genes) that are also present in primary tumours and enriched in metastatic lesions^16^. We therefore searched for metastasis-associated mutations in genes that belong to the same pathway but do not fall in recognized oncogenes.

## Results and Discussion

All the surgical specimens employed in this study were collected from consenting patients at Istituto Neurologico “Carlo Besta”, Milan, Italy, European Institute of Oncology, Milan, Italy and Erasmus University Medical Center, Rotterdam, The Netherlands.

We performed whole-exome sequencing (WES) (Illumina Hiseq. 2000 platform with 101 bp paired-end reads) of primary breast tumour and BMs from 10 patients (Supplementary Table S1a). For 7 patients we could perform WES of the matched normal brain tissue while for 3 patients (S_2, S_4 and S_9) the normal sample was unavailable. To identify metastasis-specific mutations, we compared each metastasis sample to the corresponding tumour DNA and to the normal DNA, when available. We identified somatic mutations, including single nucleotide variants (SNVs, using MuTect^17^) and small insertions and deletions (indels, using Indelocator), present in each primary BC and in the corresponding metastasis. Since we analysed somatic mutations in multiple samples for the same patient, to increase sensitivity in mutation-calling we merged the mutations identified in each sample (i.e. SNVs and indels called in the metastasis sample and tumour sample compared to the matched normal sample), and we counted the number of alternative and reference reads supporting the mutations in all the samples. Duplicated reads were not considered in this calculation. In addition, we selected variants that were present only in the metastasis, or with a variant allele frequency (VAF) significantly higher in the metastasis compared to the matched primary tumour (Fisher’s exact test corrected for FDR, values in Supplementary Table S2). The availability of normal samples allowed us to assess which somatic mutations were present in both primary and metastasis samples at higher frequency in the metastasis compared to the primary sample. Considering non-synonymous SNVs occurring with an allelic frequency of at least 5% (corresponding to a fraction of mutated cells of 10% or more, assuming the majority of mutations to be heterozygous), we identified 68 indels and 831 metastasis-specific SNVs. Of the 686 mutated genes, 78 were found in at least 2 patients, and among these 25 in at least 3 patients (Supplementary Table S2). Eight genes (*CD46*, *F5*, *C1QB*, *C5*, *FGA*, *SERPINA1*, *VWF*, *SERPINA10*) (seven with a unique mutation) found mutated in seven patients belong to the complement and coagulation cascade pathway (see Supplementary Figure S1). We found this pathway to be ranked as the third highlighted pathway and the first “not cancer” pathway as a result of analysis with Enrichr, an enrichment analysis web-based tool *((http://amp*.*pharm*.*mssm*.*edu/Enrichr/;* Supplementary Table S4).

Recent studies have shown that plasmin from the reactive brain stroma, which is lethal to cancer cells passing through the blood-brain-barrier, acts as a natural defence against metastatic invasion, and plasminogen activator (PA)-inhibitor serpins in cancer cells sustain the mechanism for the metastatic colonization of the brain^18^. These findings suggest that mutations in serpins and possibly other members of the coagulation cascade are critical to the formation of BMs. Interestingly, loss of function of one of these genes, *FGA*, is described as driving metastasis *in vivo* ^19^.

**Figure 1:**
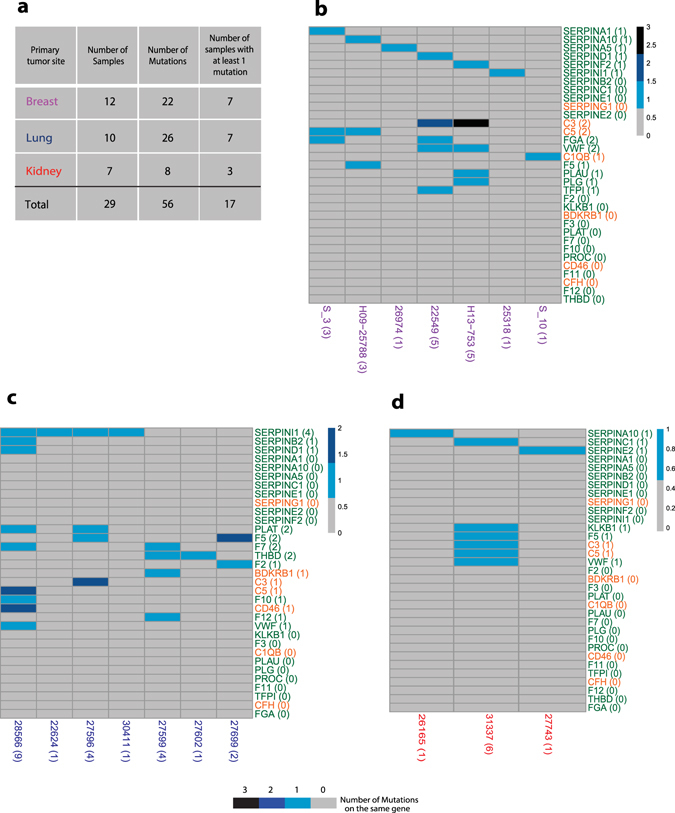
Distribution of somatic specific mutations. (**a**) Summary table of our 29 samples, including number of subjects per tumour type, total number of mutations found in coagulation and complement pathways, and number of unique genes mutated. (**b**–**d**) Oncoprint plots showing 33 genes belonging to the coagulation (26 genes, in green) and complement (7 genes, in orange) pathways on the rows and samples from three different primary tumours on the columns, (**b**) breast, (**c**) lung and (**d**) kidney respectively (17 out of 29 patients have at least one mutation on these pathways). Numbers in parenthesis represents the number of samples mutated on the specific gene (rows) and the number of mutated genes per sample (columns). Scales of blue indicates if a sample harbors one or more mutations on the same gene.

To validate the metastasis-specific mutations identified through the whole-exome sequencing analysis, we investigated the mutational status of 33 genes belonging to the complement and coagulation cascade pathway in an independent panel of BMs using the MiSeq sequencing system. We included in our panel the serpin-family genes (SFGs) since they have been previously described as relevant for the metastatization process^18^ and all the other genes belonging to the coagulation pathway that crosstalk with SFGs (see Supplementary Figure S1). Few genes (F8, F9 and F13) were excluded because the sequences were highly repetitive causing errors in the sequencing output, while few others (F2R, PLAUR, A2M, PROS1) were left out because these are genes involved in other signalling pathways. For the complement cascade, we included in the examination only upstream gene regulators, besides those genes found mutated with WES. We analysed 29 BMs derived from lung (n = 10), kidney (n = 7) and breast cancer (n = 12) (Fig. 1; Supplementary Table S1b). Six of the BC samples belong to samples previously analysed with WES (*i*.*e*. S_3, S_6, S_7, S_8, S_11, S_10). Mutations in the *F5* gene found in two patients were not validated with the Illumina MiSeq sequencing system described here. We identified 56 SNVs in 17 out of 29 BM samples distributed in 70% of patients with primary lung tumour, 58% of patients with breast primary tumour and 43% of patients with kidney primary tumour (Fig. 1a; Supplementary Table S3).

We analysed public gene mutation profiles of primary tumours coming from the cBioPortal^20, 21^ from distinct organs, and we found that the frequency of samples with at least one of the complement and coagulation pathway genes mutated was significantly lower than in BMs (12% in breast cBioPortal samples against 58% in our breast samples, 12% in kidney against 43% and 40% in lung cancer against 70%; Table 1 and in Supplementary Table S5). There is recent data showing that clinically actionable alterations in BMs are often not present in primary tumours. Such mutations may represent *de novo* alterations in BMs characteristic of the ancestral precursor of the metastatic process^16^. Among the 33 genes analysed in BMs, 27 genes were found mutated at least once (Fig. 1b–d; Supplementary Figure S1). The distribution of the mutated serpins among the samples shows a mutually exclusive pattern in breast (Fig. 1b) and in kidney (Fig. 1d). Among the 8 samples with a single mutated gene in the coagulation and complement cascade pathways, 6 had a mutation on a serpin gene, suggesting a possible self-sustainability of serpins for the entire pathway. Among SFGs, *SERPINI1* is the most frequently mutated gene, and the conserved residue S88 mutated to phenylalanine in two of our patients is the same residue mutated to alanine through a mutagenesis assay that results in an alteration of the *SERPINI1*-tPA inhibitory complex^22^.

**Table 1:** Comparison of the frequency of mutations in the coagulation and complement pathway between primary tumours from TCGA-database and brain metastases of our cohort.

TOTAL	Brain Metastasis	TCGA_Primary	OR	Permutation Test P-value		Total			
*Mutated*	17	578	5,796691	3,70E-06	*Number of samples*	2945			
*Not_Mutated*	12	2367			*Mutated Samples*	578			
*Frequency*	0,586206897	0,196264856			*Frequency*	0,196265			
**BREAST**	**Brain Metastasis**	**TCGA_Primary**	**OR**	**Permutation Test P-value**		**brca**	**Total**		
*Mutated*	7	155	9,932555	1,82E-04	*Number of samples*	1258	1258		
*Not_Mutated*	5	1103			*Mutated Samples*	155	155		
*Frequency*	0,583333333	0,123211447			*Frequency*	0,123211	0,1232114		
**LUNG**	**Brain Metastasis**	**TCGA_Primary**	**OR**	**Permutation Test P-value**		**luad**	**lusc**	**sclc**	**Total**
*Mutated*	7	313	3,498197	5,34E-02	*Number of samples*	447	178	158	783
*Not_Mutated*	3	470			*Mutated Samples*	165	79	69	313
*Frequency*	0,7	0,399744572			*Frequency*	0,369127	0,443820	0,436709	0,399745
**KIDNEY**	**Brain Metastasis**	**TCGA_Primary**	**OR**	**Permutation Test P-value**		**kich**	**kirc**	**kirp**	**Total**
*Mutated*	3	110	5,396801	4,25E-02	*Number of samples*	66	556	282	904
*Not_Mutated*	4	794			*Mutated Samples*	9	81	20	110
*Frequency*	0,428571429	0,121681416			*Frequency*	0,136364	0,145683	0,070922	0,121681

We compare frequencies of mutations based on the origin of primary tumours (breast, lung and kidney). Mutational profile of these tumours, analysed according to the pathways, indicates that coagulation and complement cascade genes carry mutations at frequency significantly lower than brain metastases in each of the 3 different datasets (breast, lung and kidney cancer).

OR: odds ratio

brca: breast carcinoma; luad: lung adenocarcinoma; lusc: lung squamous cell carcinoma; sclc: small cell lung cancer; kich: kidney chromophobe; kirc: kidney renal clear cell carcinoma; kirp: kidney renal papillary cell.

Despite the existence of several targeted therapies toward pivotal pathways in BC, the effect of drugs acting on the coagulation pathway in BM establishment and development is still unexplored. Our results suggest that the alteration of this pathway, even with a mutation of a single gene, could be a key step in the establishment of BMs. In fact, hypercoagulable state (and disorganized angiogenesis) is closely linked to tumour progression and metastatic potential, and a considerable number of clinical trials are focused on the effects of anticoagulant (and antiangiogenic) drugs on survival in cancer patients^23^.

After invading the bloodstream, tumour cells need to overcome the stress associated with circulatory passage, circulation patterns and extravasation barriers to invade, seed and form metastases^24^. The present findings support further investigations aiming at the candidacy of serpins and other molecules that take part in blood coagulation for future anti-metastatic treatment. We specifically looked at “druggable” mutated serpins since the efficacy of drug-like anti-cancer compounds in modulating serpin family members has already been shown^25, 26^. In particular, molecules targeting proteins of the serpin family may be applied to the management of patients with brain metastases.

## Methods

### Identification of metastatic-specific mutations by whole-exome sequencing

The whole procedure was carried out using experimental protocols approved by the institutional review board of the European Institute of Oncology, Milan, Italy. We confirmed that all methods were performed in accordance with the relevant guidelines and regulations and that informed consents were collected from patients in the Department of Neurosurgery at IRCCS Foundation Neurological Institute “Carlo Besta”, Milan, Italy, and from the Erasmus University Medical Center, Rotterdam, The Netherlands, under the corresponding research ethics committee approval.

We performed whole-exome sequencing (WES) on the Illumina HiSeq. 2000 platform with 101 bp paired-end reads of primary breast tumour and BMs from 10 patients. DNA of patients’ samples was extracted from paraffin-embedded (FFPE) tissues and prepared using the Qiagen DNeasy Blood & Tissue Kit, fragmented and used for Illumina Truseq library construction. To extract DNA, normal and tumoural tissue 3-4 0.6 mm core punch biopsies were prepared. Cores were chosen primarily based on sample morphology, observing hematoxylin and eosin staining: patients’ specimens were fixed in Carnoy’s solution, dehydrated, paraffin-embedded and sectioned at 2 μm according to established procedures. Slides were then stained in Carazzi hematoxylin solution, rinsed in running tap water and counterstained in eosin solution. Metastatic tissue was sharply defined and the adjacent nervous tissue contained unequivocal normal tissue (see Supplementary Figure S2)^27^. Patient normal DNA from blood was unavailable due to patients’ deaths.

Exome-capture was carried out for all the samples using the SureSelectXT Human All Exon Kit, according to the manufacturer’s instructions (Agilent Technologies, Santa Clara, CA). Alignment to the reference genomes (hg19) was performed using Burrows Wheeler Aligner (BWA)^19^. We performed the following NGS data pre-processing steps, according to GATK best practices^20^: local realignment, duplicate marking and base quality recalibration. SNVs were identified using MuTect^10^ and indels were identified using Indelocator (also part of GATK distribution), respectively. The identified variants were functionally annotated using ANNOVAR^28^, which annotates the mutations using publicly available databases (e.g. dbSNP^29^ and 1000 Genomes^30^) and identifies non-synonymous mutations. The frequency of each mutation was calculated by dividing the number of reads supporting the variant to the total coverage at the variant site.

To the identified SNVs and indels, we applied additional filters to ensure more reliable calls: i) a minimum coverage of 10 reads for both normal and tumour samples; ii) a minimum read depth of 5 for the alternate variant allele in at least one of the matched samples; iii) a variant allele frequency of at least 5% over all the reads covering the position. We excluded from further analysis variants in non-coding regions, synonymous variants, variants present in highly repetitive regions or variants annotated to be present in ExAC^31^, ESP (http://evs.gs.washington.edu/EVS) and 1000 Genomes of European origin with minor allele frequency (MAF) > 0.01.

### Identifications of variants in genes belonging to the coagulation and complement pathway by Illumina Miseq

The Illumina MiSeq sequencing system was used to identify the variants present in selected genes in additional patients. Clinical characteristics of this independent cohort of patients are reported in Supplementary Table S1b. We also included in this analysis 6 samples previously analysed with WES (S_3, S_6, S_7, S_8, S_11, S_10) whose clinical characteristics are reported in Supplementary Table S1a. We used the TruSeq Custom Amplicon kit (Illumina) and designed a panel using DesignStudio covering the following selected genes: *BDKRB1*, *C1QB*, *C3*, *C5*, *CD46*, *CFH*, *F10*, *F11*, *F12*, *F2*, *F3*, *F5*, *F7*, *FGA*, *KLKB1*, *PLAT*, *PLAU*, *PLG*, *PROC*, *SERPINA1*, *SERPINA10*, *SERPINA5*, *SERPINB2*, *SERPINC1*, *SERPIND1*, *SERPINE1*, *SERPINE2*, *SERPINF2*, *SERPING1*, *SERPINI1*, *TFPI*, *THBD*, *VWF*. We performed enrichment and sequencing of the targeted regions according to the manufacturer’s instructions. We aligned sequencing data to the reference genome hg19 with BWA^32^, and we analysed them with the same pipeline used for WES data. Briefly, we carried out alignment to the reference genomes (hg19) using BWA^19^. We performed local realignment and base quality recalibration using GATK^20^. We did not perform the removal of duplicate reads because in assays using amplicon-based capture reagents, all amplification products have the same start and stop positions making it impossible to distinguish different DNA fragments from PCR duplicate reads^33^. SNVs were identified using MuTect^10^, indels with Indelocator and ANNOVAR to annotate the identified variants. We applied the same additional filters we used for WES analysis to the identified SNVs and indels.

### Testing the difference in mutation frequency between primary tumours and metastasis

We checked for a higher frequency of mutations between our BM samples and public available samples from primary tumours contained in the cBioPortal database^13, 14^ on 33 genes in the complement and coagulation pathway. The cBioPortal database comprises the entire The Cancer Genome Atlas (TCGA) and most of the International Cancer Genome Consortium (ICGC) data and other smaller studies for a total of 1258 breast cancer samples, 783 lung cancers and 904 kidney cancers. To test for a difference in frequency, we ran a permutation test to evaluate whether the frequency in BMs was consistent with a frequency calculated on a million random samples from breast, lung and kidney cBioPortal primary tumours of the same size of our BMs cohorts for each tumour type. P-value was calculated as the fraction of random samples whose frequency was below that in our BMs. This test was preferred to a more conservative Fisher’s exact test given the disproportion between our total sample size (29 samples) and the cBioPortal data (up to 1258 samples for breast cancer). Full results including the number of mutated samples for each tumour type are available in Table 1.

## Electronic supplementary material

Supplementary Information
