# Supplementary material for: Mutations targeting the coagulation pathway are enriched in brain metastases
Source: Sci Rep. 2017 Jul 26;7:6573. doi: 10.1038/s41598-017-06811-x (PMC5529435; doi:10.1038/s41598-017-06811-x)
Supplement: Supplementary file 1 — Supplementary Information [file 41598_2017_6811_MOESM1_ESM.pdf]

Supplementary Information

Mutations targeting the coagulation pathway are enriched in brain metastases

Cristina Richichi[1], Lorenzo Fornasari[1], Giorgio E.M. Melloni[2], Paola Brescia[1], Monica Patanè[3], Massimiliano Del Bene[4], Dana A.M. Mustafa[5], Johan M. Kros[5], Bianca Pollo[3], Giancarlo Pruneri[6], Angela Sciandivasci[7], Elisabetta Munzone[7], Francesco DiMeco[4,8], Pier Giuseppe Pelicci[1,9], Laura Riva[2§], Giuliana Pelicci[1,10§].

[1]Department of Experimental Oncology, European Institute of Oncology, 20139, Milan, Italy.

[2]Center for Genomic Science of IIT@SEMM, Fondazione Istituto Italiano di Tecnologia, 20139, Milan, Italy.

[3]Department of Neuropathology, IRCCS Foundation Neurological Institute "C. Besta", 20133, Milan, Italy.

[4]Department of Neurosurgery, IRCCS Foundation Neurological Institute "C. Besta", 20133, Milan, Italy.

[5]Department of Pathology, Erasmus University Medical Center, 3015 Rotterdam, The Netherlands.

[6]Division of Pathology, European Institute of Oncology, Milano, Italy; University of Milan, Breast Cancer Program, Milano, Italy.

[7]Division of Medical Senology, European Institute of Oncology, 20141, Milan, Italy.

[8]Department of Neurosurgery, Johns Hopkins University, Baltimore, MD 21218, USA.

[9]Department of Oncology and Hemato-oncology, University of Milan, 20122, Milan, Italy.

[10]Department of Translational Medicine, Piemonte Orientale University "Amedeo Avogadro", 28100 Novara, Italy.

Supplementary Figure Legend

Supplementary Figure S1

Map of the complement and coagulation cascade (Map 04610, version 4/3/09 from the Kegg pathway database, modified[1,2]). In blue, genes pertaining to the Serpin family found mutated at least once; in red, other genes of the cascade found mutated at least once. Four genes of the Serpins family were added to the original map due to recent findings (SERPINA10, SERPINB2, SERPINI1, SERPINE2)[3–6].

1. Kanehisa M, Sato Y, Kawashima M, et al. KEGG as a reference resource for gene and protein annotation. *Nucleic Acids Res*. 2016;44:D457-D462.

2. Kanehisa M, and Goto S. KEGG: Kyoto Encyclopedia of Genes and Genomes. *Nucleic Acids Res*. 2000;28: 27-30.

3. Girard TJ, Lasky NM, Tuley EA, Broze GJ Jr. Protein Z, protein Z-dependent protease inhibitor (serpinA10), and the acute-phase response. J *Thromb Haemost.* 2013;11(2):375-378. doi: 10.1111/jth.12084.

4. Vine KL, Lobov S, Indira Chandran V, et al. Improved pharmacokinetic and biodistribution properties of the selective urokinase inhibitor PAI-2 (SerpinB2) by site-specific PEGylation: implications for drug delivery. *Pharm Res*. 2015;32(3):1045-1054. doi: 10.1007/s11095-014-1517-x. Erratum in: Pharm Res. 2015 Aug;32(8):2808. PubMed PMID: 25231010

5. Valiente M, Obenauf AC, Jin X, et al. Serpins promote cancer cell survival and vascular co-option in brain metastasis. *Cell.* 2014;156(5):1002-1016. doi: 10.1016/j.cell.2014.01.040.

6. Lu CH, Lee RK, Hwu YM, et al. Involvement of the serine protease inhibitor, SERPINE2, and the urokinase plasminogen activator in cumulus expansion and oocyte maturation. *PLoS One*. 2013;8(8):e74602. doi: 10.1371/journal.pone.0074602.

Supplementary Figure S2

Representative hematoxylin and eosin images of brain metastasis derived from (a) breast primary tumour, (b) lung primary tumour and (c) kidney primary tumour. Arrows point at the metastatic tumour edge. Scale bar = 20 μm.

Supplementary Table Legends

Supplementary Table S1

Clinical data of collected patients. Surgical specimens together with clinical records were collected from consenting patients after surgery.

Supplementary Table S1a: Patients were stratified based on the area of origin, the site of metastasis appearance, and the tumour biology indicated as subtype (by immunohistochemical classification): LUMINAL A = ER/PgR-positive and HER2-negative; LUMINAL B = ER/PgR-positive and HER2-positive; Triple Negative = ER/PgR-negative and HER2-negative. The results for ER, PgR and Ki-67 were assessed as the percentage of immunoreactive cells. Only nuclear reactivity was taken into account for ER, PgR, and Ki-67 antigen, whereas only intense membrane staining in >10% of the tumour cells were scored for HER2 overexpression (3+). HER2 scores of 0 and 1+ were considered negative. In the table, the site of metastasis is also indicated.

ER, estrogen receptor; PgR, progesterone receptor; HER2+, HER-2/Neu overexpressed.

ADJ/Y=Yes: patients that underwent brain surgery and/or adjuvant therapy; ADJ/N=No: patients that did not undergo brain surgery and/or adjuvant therapy.

NA: not allocated (infiltrating and not characterized).

NV: not evaluated.

Supplementary Table S1b: In the table are reported all the clinical data accessible for the patients whose mutational status was explored using MySeq sequencing system. Information on tumour characteristics was not available for the following patients: 25299, H09-25788, H13-753. In the Notes section, therapies are reported when available.

Lung (SCLC)= Small Cell Lung Cancer; Lung (NSCLC)= Non-Small Cell Lung Cancer

Status: 1=alive, 2=dead.

Supplementary Table S2

Metastasis specific alterations detected through whole-exome sequencing

of matched primary tumours, metastasis and the breast normal tissue from 10 patients.

Patient ID: Sample identification code

Gene Symbol: Gene symbol under HGNC nomenclature

Genomic Position: mutation position in the format Chr:Start:Ref,Alt

Function: Exonic effect of the mutation on the protein

Protein Change: amino acid change under Human Genome Variation Society (hgvs) nomenclature

dbSNP142: Reference Cluster identifier (rsID) in the database of single nucleotide polymorphisms (dbSNP) version 142

MAF in ExAC: Minor Allele Frequency in Exome Aggregation Consortium

MAF in ESP eur: Minor Allele Frequency in Exome Sequencing Project of European origin

MAF in 1KG eur: Minor Allele Frequency in Exome Aggregation Consortium of European origin

VAF in Tumour: Variant Allele Frequency in the Tumour

VAF in Metastasis: Variant Allele Frequency in the Metastasis

Q-value difference in VAF: FDR corrected Fisher test on the number of Ref and Alt alleles between tumour and metastasis

Validated: indicating if the variant was validated with MiSeq screen

Supplementary Table S3

Somatic mutations identified in brain metastases of 29 patients in our validation screen on 33 genes of the complement and cascade pathway.

Patient ID: Sample identification code

Gene Symbol: Gene symbol under HGNC nomenclature

Genomic Position: mutation position in the format Chr:Start:Ref,Alt

Function: Exonic effect of the mutation on the protein

Protein Change: amino acid change under Human Genome Variation Society (hgvs) nomenclature

dbSNP142: Reference Cluster identifier (rsID) in the database of single nucleotide polymorphisms (dbSNP) version 142

MAF in ExAC: Minor Allele Frequency in Exome Aggregation Consortium

MAF in ESP eur: Minor Allele Frequency in Exome Sequencing Project of european origin

MAF in 1KG eur: Minor Allele Frequency in Exome Aggregation Consortium of european origin

VAF: Variant Allele Frequency

Supplementary Table S4

Unbiased pathway analysis using the tool Enrichr (http://amp.pharm.mssm.edu/Enrichr/#). Enrichr implements three approaches to compute enrichment: i) the Fisher's exact test; ii) a Z-score based on deviations of the Fisher's exact values ranks from expected ranks; iii) a combination of the p-

value computed using the Fisher's exact test with the Z-score of the deviation from the expected rank, by multiplying these two numbers as follows: c=log(p)·z, (where c is the combined score, p is the p-value assessed using the Fisher exact test, and z is the z-score computed by estimating the deviation from the expected rank). We uploaded in the Enrich dialog box the list of the 686 mutated genes present in Supplementary Table S2, weighting them for the mutation frequency (from 0 to 1) within the initial cohort of 10 patients.

Overlap: genes found mutated within the genes of the investigated pathway.

Genes: genes found mutated within the genes in the WES initial cohort.

Supplementary Table S5

Comparison of the frequency of mutations in the 33 genes of the coagulation and complement pathway we found mutated between ~3000 primary tumours from cBioPortal genome database (The Cancer Genome Atlas-TCGA, in blue) and brain metastases of our cohort (in red). Mutations' frequencies were matched based on the origin of primary breast, lung and kidney tumours. Mutational profile of these tumours, analyzed according to the pathways, indicates that coagulation and complement cascade genes carry mutations at frequency significantly lower than brain metastases in each of the 3 different datasets (breast, lung and kidney cancer). P-values were calculated using a permutation test to assess whether random samples from cBioPortal cohorts have an equal or higher number of mutated samples compared to the metastases of our cohort. Sample size of each permutation is equal to the corresponding metastasis cohort. In addition, a p-value that takes in consideration all the tumour types at the same time were also calculated. FDR corrected p-values are reported in the table. Significant FDR corrected p-values are highlighted in yellow.

Richichi C. et al. Supplementary Figure S1

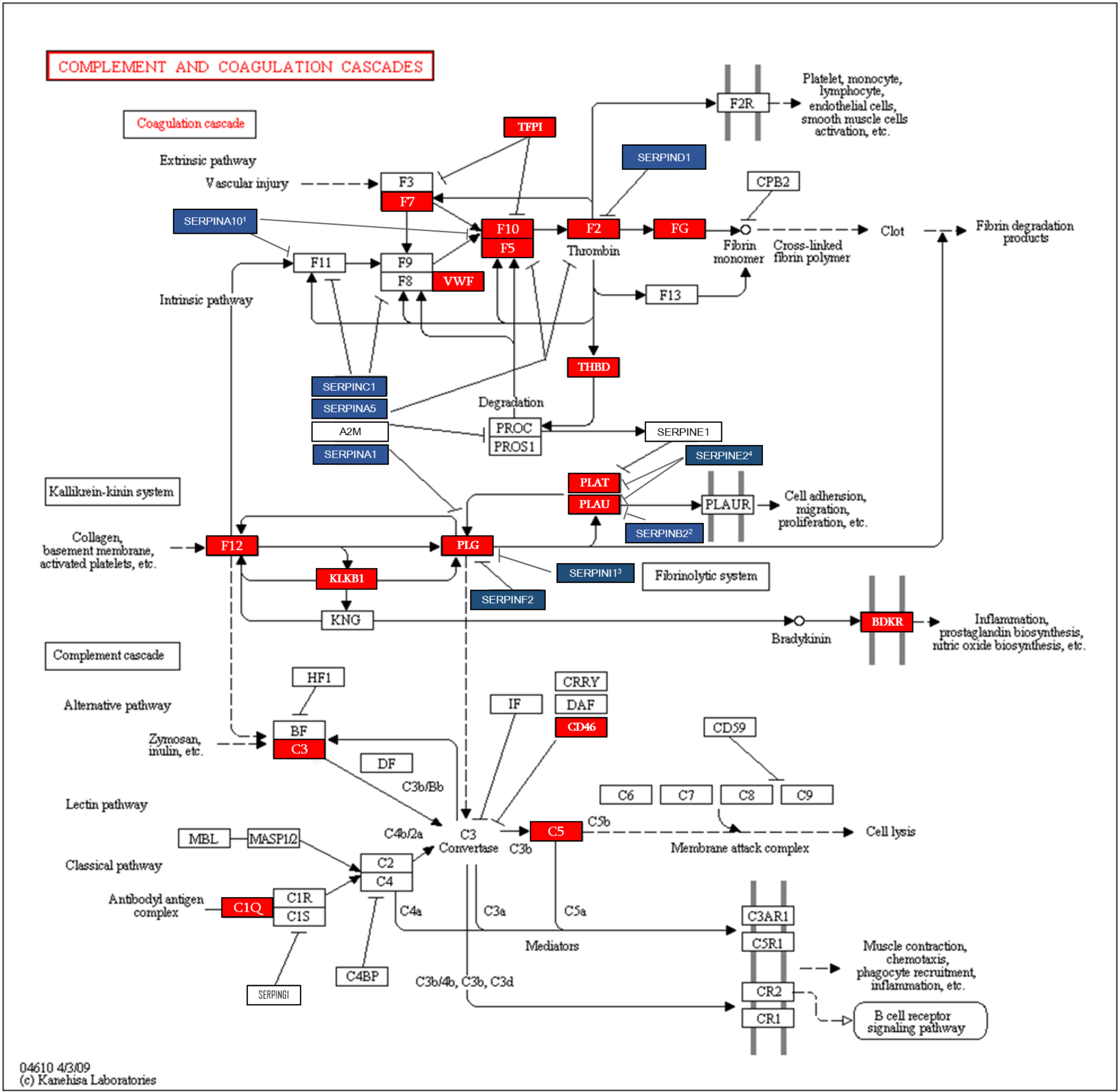

Richichi C. et al. Supplementary Figure S2

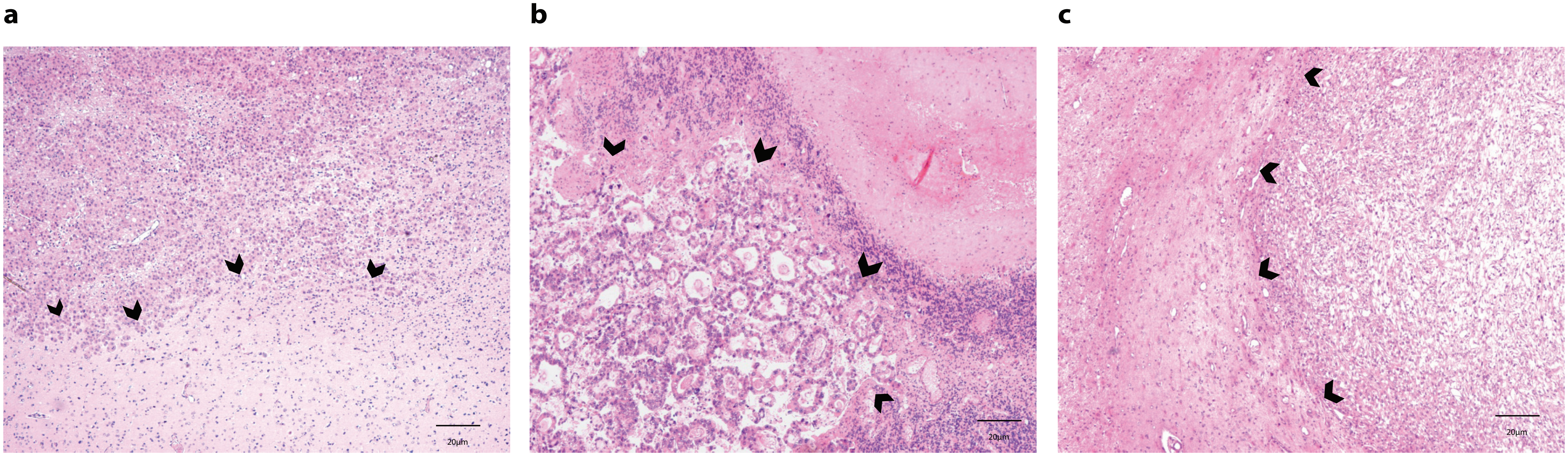

Richichi C. et al. Supplementary Table S1a

Supplementary Table S1a. Clinical characteristics **o**f patients studied through whole-exome sequencing.

Patient ID	Age	Breast Primary	ER %	PgR %	HER2+	Ki67 %	Subtype	ADJ Y/N	Site Metastasis	Brain surgery
**S_1**	51	ductal	85	2	0	45	LUMINAL B	Y	Nodes, Brain	Y
**S_2**	43	ductal	60	10	2+	40	LUMINAL B	Y	Nodes, Brain	Y
**S_3**	32	ductal	90	25	1+	36	LUMINAL B	Y	NV	Y
**S_4**	50	infiltrating carcinoma	0	0	3+	32	HER2+	Y	Nodes, Skin, Brain	Y
**S_5**	51	ductal	0	0	3+	40	HER2+	Y	Nodes, Skin, Brain	Y
**S_6**	43	ductal	90	60	0	25	LUMINAL B	Y	Brain	Y
**S_7**	46	infiltrating carcinoma	0	0	3+	31	HER2+	Y	Bone, Brain	Y
**S_8**	52	lobular	85	70	0	20	LUMINAL B	Y	Brain	Y
**S_9**	40	ductal	25	20	3+	35	HER2+	Y	Nodes, Brain	Y
**S_10**	48	ductal	0	0	3+	55	HER2+	N	Brain	Y
**S_11**	33	ductal	0	0	0	80	Triple Negative (TN)	Y	Brain	Y
**S_12**	46	mucinous	95	20	1+	4	LUMINAL A	Y	Bone, Brain	Y

Richichi C. et al. Supplementary Table S1

Supplementary Table S1b. Clinical characteristics of an independent cohort of patients investigated through the MiSeq Sequencing System.

Patient ID	Age at the time of surgery	Primary Tumor Type	Site of Brain Metastasis	Number of lesions	Survival time (months)	Status	Notes
22357	59	Lung (SCLC)	NA	Single	1	2	No chemo-radiotherapy
22549	39	Breast	cerebellum	Single	6	2	Radiotherapy
22624	63	Lung (SCLC)	NA	Single	49	2	Radiotherapy
24957	60	Lung (adenocarcinoma)	occipital lobe	Single	11	2	No chemo-radiotherapy
25318	53	Breast	occipital-parietal lobe	Multiple	23	2	Radiotherapy
25398	72	Kidney (clear cells)	cerebellum	Single	43	2	NA
26165	55	Kidney (clear cells)	temporal lobe	Single	16	2	No chemo-radiotherapy
26208	76	Kidney (clear cells)	brain parenchyma	Single	19	2	NA
26266	54	Breast	frontal lobe	Single	69	2	NA
26974	42	Breast	cerebellum; temporal lobe	Multiple	31	2	Surgeries with cyberknife tecnology
27596	69	Lung (adenocarcinoma)	parietal-occipital lobe	Single	3	2	Radiosurgery
27599	60	Lung	posterior fossa	Single	2	2	Surgery with cyberknife tecnology
27602	65	Lung	cerebellum; temporal lobe	Single	10	2	No chemo-radiotherapy
27699	53	Lung (NSCLC)	frontal lobe	Single	3	2	No chemo-radiotherapy

27743	76	Kidney (clear cells)	occipital lobe	Single	84	1	Radiotherapy
28566	63	Lung (adenocarcinoma)	cerebellum	Single	5	2	No chemo-radiotherapy
30411	69	Lung (adenocarcinoma)	frontal lobe	Single	<1	2	NA
31196	60	Kidney (clear cells)	spinal	Single	65	1	NA
31337	65	Kidney (clear cells)	frontal lobe	Single	2	2	Recurrent Metastases
32187	56	Kidney (clear cells)	occipital lobe	Single	22	2	NA

Supplementary Table S2. Metastasis specific alterations detected through whole-exome sequencing of matched primary breast tumors, brain metastasis and breast normal tissue.

Patient ID	Gene Symbol	Genomic Position	Function	Protein Change	dbSNP142	MAF in ExAC	MAF in ESP eur	MAF in 1KG eur	VAF in Tumor	VAF in Metastasis	Q-value difference in VAF	Validated
S_10	DUS1L	17:80021368:C,T	nonsynonymous SNV	p.E125K	rs76779621	5,69E-05	0	0,001	0	0,157894737	NA	
S_10	FBXO39	17:6683194:G,A	nonsynonymous SNV	p.E3K	rs368045455	1,63E-05	0,000116	0	0	0,226415094	NA	
S_10	MAGEF1	3:184429216:C,G	nonsynonymous SNV	p.E132Q	rs201193072	0,000138	0,000349	0	0	0,363636364	NA	
S_10	FAT4	4:126372561:G,A	nonsynonymous SNV	p.V3466I	rs373744467	1,63E-05	0	0	0	0,219512195	NA	
S_10	AFAP1L2	10:116068273:C,T	nonsynonymous SNV	p.D296N	rs144885661	5,69E-05	0	0	0	0,155555556	NA	
S_10	FAM208B	10:5782276:G,A	nonsynonymous SNV	p.V715M	rs377604488	4,09E-05	0	0	0	0,220264317	NA	
S_10	C17orf64	17:58506841:G,A	nonsynonymous SNV	p.R183Q	rs572279125	8,13E-06	0	0	0	0,156914894	NA	
S_10	CPT1C	19:50200652:C,T	nonsynonymous SNV	p.L71F		8,13E-06	0	0	0	0,386363636	NA	
S_10	DPCR1	6:30920879:C,G	nonsynonymous SNV	p.I1389M		8,13E-06	0	0	0	0,28125	NA	
S_10	CXorf23	X:19984291:T,G	nonsynonymous SNV	p.E173A		8,17E-06	0	0	0	0,171974522	NA	
S_10	PTPN13	4:87686563:A,C	nonsynonymous SNV	p.S1198R		8,18E-06	0	0	0	0,272727273	NA	
S_10	FAM73A	1:78245357:C,G	stopgain	p.S6X		8,20E-06	0	0	0	0,122222222	NA	
S_10	TNFRSF1A	12:6438654:C,T	nonsynonymous SNV	p.E398K		8,30E-06	0	0	0,027586207	0,301204819	3,10E-10	
S_10	ZBTB4	17:7369603:G,A	nonsynonymous SNV	p.S173F		1,63E-05	0	0	0	0,417218543	NA	
S_10	ZNF136	19:12298668:G,A	nonsynonymous SNV	p.R492Q		1,63E-05	0	0	0	0,315789474	NA	
S_10	ALMS1	2:73675816:A,C	nonsynonymous SNV	p.K720T		1,64E-05	0	0	0	0,279069767	NA	
S_10	HBS1L	6:135318032:A,C	nonsynonymous SNV	p.L241R		2,44E-05	0	0	0	0,370967742	NA	
S_10	TBC1D10B	16:30369642:G,A	nonsynonymous SNV	p.R684C		2,51E-05	0	0	0	0,070422535	NA	
S_10	MEGF6	1:3407153:G,A	nonsynonymous SNV	p.A1522V	rs201358713	3,28E-05	0	0	0	0,35	NA	
S_10	PTPN13	4:87686567:T,G	nonsynonymous SNV	p.V1199G	rs199630109	5,73E-05	0	0	0	0,272727273	NA	
S_10	IGFN1	1:201179829:G,A	nonsynonymous SNV	p.M1936I		0,000144	0	0	0,025423729	0,1328125	0,007232864	
S_10	C5	9:123739039:T,G	nonsynonymous SNV	p.N1268T		0	0	0	0	0,404761905	NA	validated
S_10	FGA	4:155507877:C,T	nonsynonymous SNV	p.G235E		0	0	0	0	0,285714286	NA	validated
S_10	SERPINA1	14:94848980:C,T	nonsynonymous SNV	p.E199K		0	0	0	0	0,333333333	NA	validated
S_10	ABCA8	17:66925760:A,C	nonsynonymous SNV	p.I294S		0	0	0	0	0,163265306	NA	
S_10	ABHD8	19:17411837:A,C	nonsynonymous SNV	p.F197V		0	0	0	0	0,329896907	NA	
S_10	ACKR3	2:237489581:A,C	nonsynonymous SNV	p.K158T		0	0	0	0	0,221662469	NA	
S_10	ACVR1B	12:52345534:G,T	stopgain	p.E3X		0	0	0	0	0,37037037	NA	
S_10	ADAT1	16:75646409:T,G	nonsynonymous SNV	p.T259P		0	0	0	0	0,213793103	NA	
S_10	AGBL2	11:47726223:A,G	nonsynonymous SNV	p.L153S		0	0	0	0	0,24	NA	
S_10	AGBL2	11:47726226:T,G	nonsynonymous SNV	p.E152A		0	0	0	0	0,291666667	NA	
S_10	AHNAK	11:62295840:C,T	nonsynonymous SNV	p.E2017K		0	0	0	0	0,171052632	NA	
S_10	AIM1L	1:26672978:G,C	nonsynonymous SNV	p.F57L		0	0	0	0	0,361111111	NA	
S_10	AKAP12	6:151672282:C,G	nonsynonymous SNV	p.T821R		0	0	0	0	0,252631579	NA	
S_10	ALDOC	17:26900614:T,G	nonsynonymous SNV	p.K342T		0	0	0	0	0,257575758	NA	
S_10	ALKBH6	19:36503961:A,C	nonsynonymous SNV	p.F57V		0	0	0	0	0,294117647	NA	
S_10	ANKRD24	19:4216306:A,C	nonsynonymous SNV	p.Q432H		0	0	0	0	0,35443038	NA	
S_10	AQR	15:35155204:C,T	nonsynonymous SNV	p.R1298K		0	0	0	0	0,2	NA	
S_10	ARAF	X:47422668:A,C	nonsynonymous SNV	p.K47T		0	0	0	0	0,333333333	NA	
S_10	ARHGEF33	2:39193198:A,C	nonsynonymous SNV	p.S799R		0	0	0	0	0,296296296	NA	
S_10	ARL6IP5	3:69153740:G,C	nonsynonymous SNV	p.E174Q		0	0	0	0	0,357142857	NA	

S_10	ARSI	5:149678033:A,C	nonsynonymous SNV	p.F152V	0	0	0	0	0,258536585	NA
S_10	ASH1L	1:155451700:A,C	nonsynonymous SNV	p.L321V	0	0	0	0	0,240384615	NA
S_10	ATHL1	11:290399:T,A	nonsynonymous SNV	p.L90H	0	0	0	0	0,134615385	NA
S_10	ATP10A	15:25924679:A,C	nonsynonymous SNV	p.L1437V	0	0	0	0	0,222972973	NA
S_10	ATPIF1	1:28564378:G,C	nonsynonymous SNV	p.L70F	0	0	0	0	0,291666667	NA
S_10	AXL	19:41743907:A,T	nonsynonymous SNV	p.E13V	0	0	0	0	0,275689223	NA
S_10	AXL	19:41759559:A,G	nonsynonymous SNV	p.E393G	0	0	0	0	0,248275862	NA
S_10	BAZ1A	14:35228019:G,C	nonsynonymous SNV	p.S1394C	0	0	0	0	0,12	NA
S_10	BCL6B	17:6927001:C,A	nonsynonymous SNV	p.P4H	0	0	0	0,029411765	0,375	2,73E-06
S_10	BCL9L	11:118771423:G,A	nonsynonymous SNV	p.S1010L	0	0	0	0	0,390804598	NA
S_10	BNC1	15:83926538:C,T	nonsynonymous SNV	p.E874K	0	0	0	0	0,196850394	NA
S_10	BRAF	7:140481472:A,T	nonsynonymous SNV	p.S446T	0	0	0	0	0,318181818	NA
S_10	BTN1A1	6:26509052:A,G	nonsynonymous SNV	p.T411A	0	0	0	0	0,211009174	NA
S_10	CA13	8:86163094:A,C	nonsynonymous SNV	p.S55R	0	0	0	0	0,159235669	NA
S_10	CACNA1A	19:13411450:T,G	nonsynonymous SNV	p.E732D	0	0	0	0	0,230769231	NA
S_10	CAMSAP2	1:200818605:T,G	nonsynonymous SNV	p.L887R	0	0	0	0	0,253521127	NA
S_10	CCDC87	11:66359931:C,G	nonsynonymous SNV	p.E186Q	0	0	0	0	0,268867925	NA
S_10	CCR6	6:167550312:A,C	nonsynonymous SNV	p.E198D	0	0	0	0	0,35	NA
S_10	CDC42BPB	14:103412925:C,T	nonsynonymous SNV	p.E1210K	0	0	0	0,042857143	0,280701754	0,003208597
S_10	CDT1	16:88871004:C,G	nonsynonymous SNV	p.P94A	0	0	0	0	0,22875817	NA
S_10	CES5A	16:55886888:T,G	nonsynonymous SNV	p.K393T	0	0	0	0	0,333333333	NA
S_10	CHST4	16:71570638:T,G	nonsynonymous SNV	p.L20V	0	0	0	0	0,182481752	NA
S_10	CLCN2	3:184071058:C,T	nonsynonymous SNV	p.E626K	0	0	0	0	0,317647059	NA
S_10	CMYA5	5:79028989:A,C	nonsynonymous SNV	p.E1467D	0	0	0	0	0,304347826	NA
S_10	COL12A1	6:75833738:G,C	nonsynonymous SNV	p.T1102S	0	0	0	0	0,368421053	NA
S_10	COL6A6	3:130287253:G,A	nonsynonymous SNV	p.D736N	0	0	0	0	0,363636364	NA
S_10	CSMD2	1:34003129:A,C	nonsynonymous SNV	p.F3094V	0	0	0	0	0,351351351	NA
S_10	CYLC1	X:83128293:A,C	nonsynonymous SNV	p.K193Q	0	0	0	0	0,277777778	NA
S_10	DAPK1	9:90301555:G,C	nonsynonymous SNV	p.G772R	0	0	0	0	0,275362319	NA
S_10	DDX18	2:118586573:C,T	stopgain	p.Q562X	0	0	0	0	0,243243243	NA
S_10	DENND2D	1:111741299:G,C	nonsynonymous SNV	p.F100L	0	0	0	0	0,45	NA
S_10	DENND4A	15:66030085:G,A	nonsynonymous SNV	p.R334C	0	0	0	0,010204082	0,303030303	5,26E-05
S_10	DISC1	1:231829952:A,T	nonsynonymous SNV	p.M150L	0	0	0	0	0,300275482	NA
S_10	DLC1	8:12958079:C,G	nonsynonymous SNV	p.Q78H	0	0	0	0	0,46835443	NA
S_10	DNAH9	17:11593362:T,G	nonsynonymous SNV	p.L1408R	0	0	0	0	0,377777778	NA
S_10	DNAH9	17:11666783:A,C	nonsynonymous SNV	p.E2341A	0	0	0	0	0,222222222	NA
S_10	DNMT1	19:10246804:C,T	nonsynonymous SNV	p.G1534D	0	0	0	0	0,336633663	NA
S_10	DST	6:56504943:G,C	nonsynonymous SNV	p.Q293E	0	0	0	0	0,416666667	NA
S_10	DTHD1	4:36292024:G,T	nonsynonymous SNV	p.G16V	0	0	0	0	0,538461538	NA
S_10	DUS1L	17:80016233:C,G	nonsynonymous SNV	p.E422D	0	0	0	0	0,131428571	NA
S_10	EGFL6	X:13626496:T,G	nonsynonymous SNV	p.F237V	0	0	0	0	0,121107266	NA
S_10	EHMT2	6:31857374:T,G	nonsynonymous SNV	p.E347D	0	0	0	0	0,142857143	NA
S_10	ELAVL2	9:23692776:C,A	nonsynonymous SNV	p.A274S	0	0	0	0	0,307692308	NA
S_10	ENPP2	8:120583025:A,C	nonsynonymous SNV	p.L613V	0	0	0	0	0,236363636	NA
S_10	EP400	12:132466742:T,G	nonsynonymous SNV	p.L550V	0	0	0	0	0,234042553	NA
S_10	ERCC6L	X:71426562:T,A	nonsynonymous SNV	p.K685N	0	0	0	0	0,304347826	NA
S_10	EVC2	4:5696133:A,C	nonsynonymous SNV	p.L47V	0	0	0	0	0,5	NA
S_10	FAM160B2	8:21960061:G,C	nonsynonymous SNV	p.R672T	0	0	0	0	0,120689655	NA

S_10	FAM160B2	8:21960068:G,T	nonsynonymous SNV	p.Q674H	0	0	0	0	0,151515152	NA
S_10	FAM189B	1:155223663:C,G	nonsynonymous SNV	p.R148P	0	0	0	0	0,344978166	NA
S_10	FAM91A1	8:124789520:G,A	nonsynonymous SNV	p.E109K	0	0	0	0	0,147058824	NA
S_10	FBRSL1	12:133067340:C,T	nonsynonymous SNV	p.R62C	0	0	0	0	0,421052632	NA
S_10	FBXO6	1:11731984:G,C	NA	NA	0	0	0	0	0,170212766	NA
S_10	FBXO6	1:11732022:G,C	nonsynonymous SNV	p.E151Q	0	0	0	0	0,173913043	NA
S_10	FKBP15	9:115932803:T,G	nonsynonymous SNV	p.K922T	0	0	0	0	0,37254902	NA
S_10	FLG2	1:152327695:G,C	nonsynonymous SNV	p.S856C	0	0	0	0	0,21719457	NA
S_10	FMNL1	17:43323878:A,C	nonsynonymous SNV	p.K1073T	0	0	0	0	0,355731225	NA
S_10	FRYL	4:48591827:T,G	nonsynonymous SNV	p.E525D	0	0	0	0	0,133333333	NA
S_10	FSIP2	2:186669686:C,T	nonsynonymous SNV	p.S5307L	0	0	0	0	0,277777778	NA
S_10	GCK	7:44193023:C,T	nonsynonymous SNV	p.D29N	0	0	0	0	0,20754717	NA
S_10	GCN1L1	12:120600877:A,C	nonsynonymous SNV	p.L679V	0	0	0	0	0,29707113	NA
S_10	GLMN	1:92737128:C,G	nonsynonymous SNV	p.E273Q	0	0	0	0	0,2	NA
S_10	GRIN2C	17:72838941:G,T	stopgain	p.S1112X	0	0	0	0	0,171052632	NA
S_10	GRM5	11:88386415:T,G	nonsynonymous SNV	p.Q356H	0	0	0	0	0,35	NA
S_10	GSDMB	17:38064462:G,A	nonsynonymous SNV	p.H223Y	0	0	0	0,000707714	0,117892977	6,30E-57
S_10	HAGHL	16:777579:G,A	nonsynonymous SNV	p.E24K	0	0	0	0	0,284552846	NA
S_10	HECW1	7:43484575:G,A	nonsynonymous SNV	p.E602K	0	0	0	0	0,299465241	NA
S_10	HIST2H2BE	1:149857978:G,C	nonsynonymous SNV	p.F71L	0	0	0	0	0,153061224	NA
S_10	HK1	10:71139737:A,C	nonsynonymous SNV	p.N384T	0	0	0	0	0,256880734	NA
S_10	HLX	1:221057702:G,C	nonsynonymous SNV	p.E375Q	0	0	0	0	0,2	NA
S_10	HMCN2	9:133067085:C,A	unknown	NA	0	0	0	0	0,291666667	NA
S_10	HMCN2	9:133303846:T,G	unknown	NA	0	0	0	0	0,262626263	NA
S_10	HS3ST2	16:22825968:C,T	stopgain	p.Q13X	0	0	0	0	0,274193548	NA
S_10	HSD11B1L	19:5686505:G,C	nonsynonymous SNV	p.E8Q	0	0	0	0	0,27027027	NA
S_10	IGFN1	1:201182339:G,C	nonsynonymous SNV	p.R2773T	0	0	0	0	0,419117647	NA
S_10	IKBKB	8:42176799:T,A	nonsynonymous SNV	p.L457H	0	0	0	0	0,260869565	NA
S_10	IL18R1	2:103006629:T,G	nonsynonymous SNV	p.L199V	0	0	0	0	0,333333333	NA
S_10	ITIH5	10:7618965:C,A	stopgain	p.E263X	0	0	0	0	0,216981132	NA
S_10	KCNMA1	10:78778793:T,C	nonsynonymous SNV	p.S665G	0	0	0	0	0,409090909	NA
S_10	KDM4B	19:5143984:G,A	nonsynonymous SNV	p.V853M	0	0	0	0,009345794	0,231213873	2,49E-07
S_10	KIAA1462	10:30318136:T,G	nonsynonymous SNV	p.Q314P	0	0	0	0	0,35	NA
S_10	KIFC3	16:57794274:C,T	nonsynonymous SNV	p.E624K	0	0	0	0	0,220994475	NA
S_10	KLF11	2:10187820:C,T	nonsynonymous SNV	p.T102I	0	0	0	0,01369863	0,187793427	3,50E-09
S_10	KLHL17	1:900426:A,C	nonsynonymous SNV	p.E595A	0	0	0	0	0,407407407	NA
S_10	KRR1	12:75902137:T,C	nonsynonymous SNV	p.S59G	0	0	0	0	0,194444444	NA
S_10	KRT78	12:53233659:T,G	nonsynonymous SNV	p.K276T	0	0	0	0,003745318	0,273159145	3,56E-25
S_10	KRTAP10-11	21:46067102:C,T	nonsynonymous SNV	p.P243S	0	0	0	0	0,330827068	NA
S_10	LAMA5	20:60905971:T,G	nonsynonymous SNV	p.K1227T	0	0	0	0	0,140449438	NA
S_10	LENG9	19:54973587:G,A	nonsynonymous SNV	p.R375W	0	0	0	0	0,1875	NA
S_10	LMCD1	3:8578903:A,T	nonsynonymous SNV	p.E55V	0	0	0	0	0,319148936	NA
S_10	LMNB2	19:2435094:A,C	nonsynonymous SNV	p.F254V	0	0	0	0	0,383458647	NA
S_10	LNX1	4:54362371:T,G	nonsynonymous SNV	p.E294A	0	0	0	0	0,182539683	NA
S_10	LRBA	4:151727485:G,A	nonsynonymous SNV	p.A1819V	0	0	0	0	0,405405405	NA
S_10	LTBP3	11:65314018:C,T	nonsynonymous SNV	p.E750K	0	0	0	0	0,292682927	NA
S_10	LY75,LY75-CD302	2:160697324:G,C	nonsynonymous SNV	p.I1141M	0	0	0	0	0,269230769	NA

S_10	MAK	6:10775625:G,T	nonsynonymous SNV	p.N511K	0	0	0	0	0,25	NA
S_10	MBD5	2:149247493:A,C	nonsynonymous SNV	p.D1198A	0	0	0	0	0,355263158	NA
S_10	MEGF8	19:42866575:G,A	nonsynonymous SNV	p.E1895K	0	0	0	0	0,196428571	NA
S_10	MERTK	2:112785984:C,T	nonsynonymous SNV	p.S848L	0	0	0	0	0,264150943	NA
S_10	MKI67	10:129905531:A,C	nonsynonymous SNV	p.F1165V	0	0	0	0	0,340909091	NA
S_10	MOGS	2:74689799:C,G	nonsynonymous SNV	p.E373Q	0	0	0	0	0,275280899	NA
S_10	MRC2	17:60769762:G,A	nonsynonymous SNV	p.E1464K	0	0	0	0	0,168316832	NA
S_10	MUC2	11:1103263:C,G	nonsynonymous SNV	p.I2667M	0	0	0	0	0,644104803	NA
S_10	MUC21	6:30954260:G,C	nonsynonymous SNV	p.S103T	0	0	0	0	0,242038217	NA
S_10	MUTYH	1:45800080:T,G	nonsynonymous SNV	p.K47T	0	0	0	0	0,381578947	NA
S_10	NBEAL1	2:204073911:C,T	stopgain	p.Q2522X	0	0	0	0	0,326530612	NA
S_10	NCOA2	8:71128955:G,C	nonsynonymous SNV	p.S9C	0	0	0	0	0,079545455	NA
S_10	NCOA6	20:33328299:G,C	nonsynonymous SNV	p.L1921V	0	0	0	0	0,183673469	NA
S_10	NELFE	6:31926155:C,G	nonsynonymous SNV	p.K23N	0	0	0	0	0,234375	NA
S_10	NLRP5	19:56511105:G,T	nonsynonymous SNV	p.G5V	0	0	0	0	0,310344828	NA
S_10	NMT1	17:43175832:G,C	nonsynonymous SNV	p.E266Q	0	0	0	0	0,357142857	NA
S_10	OPRM1	6:154412122:A,C	nonsynonymous SNV	p.T127P	0	0	0	0	0,325	NA
S_10	OR4S2	11:55418892:G,C	nonsynonymous SNV	p.E171D	0	0	0	0	0,236842105	NA
S_10	OR5A2	11:59190371:G,C	stopgain	p.S19X	0	0	0	0	0,280701754	NA
S_10	OR6Y1	1:158517628:A,C	nonsynonymous SNV	p.F90V	0	0	0	0	0,325	NA
S_10	OR7A17	19:14991431:G,C	stopgain	p.S246X	0	0	0	0	0,323529412	NA
S_10	OTOG	11:17627445:T,G	nonsynonymous SNV	p.F1319V	0	0	0	0	0,349056604	NA
S_10	OTOG	11:17660009:T,G	nonsynonymous SNV	p.F2615V	0	0	0	0	0,316129032	NA
S_10	P2RY4	X:69478402:G,T	nonsynonymous SNV	p.S358Y	0	0	0	0	0,374045802	NA
S_10	PCDHGA11	5:140802623:T,G	nonsynonymous SNV	p.L610R	0	0	0	0	0,180555556	NA
S_10	PDE4D	5:58511639:T,G	nonsynonymous SNV	p.K204T	0	0	0	0	0,5	NA
S_10	PDYN	20:1961103:A,C	nonsynonymous SNV	p.L211V	0	0	0	0	0,050652341	NA
S_10	PDYN	20:1963693:A,C	nonsynonymous SNV	p.L13R	0	0	0	0	0,067398119	NA
S_10	PEAK1	15:77473526:T,C	nonsynonymous SNV	p.E248G	0	0	0	0	0,235294118	NA
S_10	PFKFB3	10:6262824:A,G	nonsynonymous SNV	p.K256R	0	0	0	0	0,313186813	NA
S_10	PGS1	17:76399701:C,G	nonsynonymous SNV	p.I311M	0	0	0	0	0,13372093	NA
S_10	PHF3	6:64421998:C,G	stopgain	p.S1505X	0	0	0	0	0,454545455	NA
S_10	PKDREJ	22:46653858:T,G	nonsynonymous SNV	p.I1788L	0	0	0	0	0,254901961	NA
S_10	PKHD1L1	8:110530654:T,C	nonsynonymous SNV	p.L3983P	0	0	0	0	0,130952381	NA
S_10	PLCG2	16:81819698:A,C	nonsynonymous SNV	p.K35T	0	0	0	0	0,201058201	NA
S_10	PLK2	5:57751485:C,G	nonsynonymous SNV	p.Q502H	0	0	0	0	0,225806452	NA
S_10	PPM1E	17:57057856:A,C	nonsynonymous SNV	p.S578R	0	0	0	0	0,168539326	NA
S_10	PPP1R37	19:45645621:A,C	nonsynonymous SNV	p.N260T	0	0	0	0	0,232472325	NA
S_10	PRUNE2	9:79320440:C,G	nonsynonymous SNV	p.W2250C	0	0	0	0	0,3	NA
S_10	RAD51D	17:33430547:C,T	nonsynonymous SNV	p.G86E	0	0	0	0	0,070895522	NA
S_10	RASGRP2	11:64503133:T,G	nonsynonymous SNV	p.T393P	0	0	0	0	0,272727273	NA
S_10	RASSF7	11:562264:G,C	nonsynonymous SNV	p.E104Q	0	0	0	0	0,374100719	NA
S_10	RFXAP	13:37393562:C,T	nonsynonymous SNV	p.A23V	0	0	0	0	0,361702128	NA
S_10	RIMS2	8:104897929:C,G	nonsynonymous SNV	p.H176D	0	0	0	0	0,072100313	NA
S_10	RIPK3	14:24807689:A,C	nonsynonymous SNV	p.L186V	0	0	0	0	0,291208791	NA
S_10	RNF8	6:37349042:G,T	nonsynonymous SNV	p.L451F	0	0	0	0	0,304347826	NA
S_10	ROCK1	18:18548767:T,G	nonsynonymous SNV	p.K990T	0	0	0	0	0,25	NA
S_10	RPRD2	1:150445664:T,G	nonsynonymous SNV	p.L1388V	0	0	0	0	0,27972028	NA

S_10	RPS24	10:79795471:T,G	nonsynonymous SNV	p.L91R	0	0	0	0	0,344827586	NA
S_10	RPS4X	X:71493781:T,G	nonsynonymous SNV	p.K134N	0	0	0	0	0,304347826	NA
S_10	RRM2	2:10262891:C,A	nonsynonymous SNV	p.S49Y	0	0	0	0	0,387096774	NA
S_10	RSF1	11:77411893:T,A	nonsynonymous SNV	p.Q794L	0	0	0	0	0,148387097	NA
S_10	RYR1	19:38964197:C,T	nonsynonymous SNV	p.P1316S	0	0	0	0	0,31092437	NA
S_10	S100A3	1:153520843:T,G	nonsynonymous SNV	p.K40T	0	0	0	0	0,232365145	NA
S_10	SAMD14	17:48195053:C,G	nonsynonymous SNV	p.D73H	0	0	0	0	0,285714286	NA
S_10	SDAD1	4:76897116:C,G	nonsynonymous SNV	p.K109N	0	0	0	0	0,294117647	NA
S_10	SESN3	11:94908721:C,T	nonsynonymous SNV	p.E306K	0	0	0	0	0,259259259	NA
S_10	SETBP1	18:42530467:A,C	nonsynonymous SNV	p.S388R	0	0	0	0,0078125	0,377358491	4,18E-10
S_10	SH2B3	12:111856488:A,C	nonsynonymous SNV	p.K180T	0	0	0	0	0,243243243	NA
S_10	SLC12A2	5:127493832:G,C	nonsynonymous SNV	p.L817F	0	0	0	0	0,24137931	NA
S_10	SLC15A2	3:121650519:G,C	nonsynonymous SNV	p.K535N	0	0	0	0	0,366666667	NA
S_10	SLC23A3	2:220027102:C,T	nonsynonymous SNV	p.D369N	0	0	0	0	0,433333333	NA
S_10	SLC25A46	5:110097463:T,G	nonsynonymous SNV	p.L413R	0	0	0	0	0,625	NA
S_10	SLC4A11	20:3209901:A,C	nonsynonymous SNV	p.F636V	0	0	0	0	0,26779661	NA
S_10	SLC4A2	7:150763800:G,A	nonsynonymous SNV	p.E250K	0	0	0	0	0,418803419	NA
S_10	SPTBN4	19:41063233:A,G	nonsynonymous SNV	p.E541G	0	0	0	0	0,232704403	NA
S_10	SRMS	20:62172639:G,T	nonsynonymous SNV	p.A397E	0	0	0	0	0,153846154	NA
S_10	STC1	8:23702453:T,G	nonsynonymous SNV	p.S192R	0	0	0	0	0,333333333	NA
S_10	SYPL2	1:110018204:T,C	nonsynonymous SNV	p.L44P	0	0	0	0,016129032	0,290322581	4,58E-05
S_10	TACC1	8:38677415:C,G	nonsynonymous SNV	p.S23C	0	0	0	0	0,358490566	NA
S_10	TAF1	X:70683739:G,T	nonsynonymous SNV	p.G1844V	0	0	0	0	0,178571429	NA
S_10	TBC1D9B	5:179305341:C,T	nonsynonymous SNV	p.A584T	0	0	0	0	0,212121212	NA
S_10	TENM3	4:183710256:G,A	nonsynonymous SNV	p.R1772K	0	0	0	0	0,25	NA
S_10	TENM3	4:183710309:G,A	nonsynonymous SNV	p.D1790N	0	0	0	0	0,216666667	NA
S_10	TGM1	14:24724255:G,A	nonsynonymous SNV	p.S617L	0	0	0	0	0,25	NA
S_10	TMC3	15:81633809:A,G	nonsynonymous SNV	p.L589P	0	0	0	0	0,285714286	NA
S_10	TMEM132E	17:32964422:A,C	nonsynonymous SNV	p.K709T	0	0	0	0	0,058921162	NA
S_10	TMEM68	8:56675361:A,C	nonsynonymous SNV	p.L53R	0	0	0	0	0,107692308	NA
S_10	TMEM74B	20:1161790:T,G	nonsynonymous SNV	p.E158A	0	0	0	0	0,053421634	NA
S_10	TMOD1	9:100308498:A,C	nonsynonymous SNV	p.K51T	0	0	0	0	0,333333333	NA
S_10	TRIM25	17:54969212:A,C	nonsynonymous SNV	p.L581R	0	0	0	0	0,20661157	NA
S_10	TTC13	1:231060653:C,T	nonsynonymous SNV	p.R499Q	0	0	0	0	0,625	NA
S_10	TTN	2:179446399:T,G	nonsynonymous SNV	p.D13134A	0	0	0	0	0,261904762	NA
S_10	UFM1	13:38928433:A,C	nonsynonymous SNV	p.E37D	0	0	0	0	0,333333333	NA
S_10	UHRF1BP1	6:34824584:C,T	stopgain	p.R437X	0	0	0	0	0,176470588	NA
S_10	USE1	19:17330074:G,C	nonsynonymous SNV	p.D159H	0	0	0	0	0,284615385	NA
S_10	USP2	11:119230323:C,G	nonsynonymous SNV	p.L48F	0	0	0	0	0,170731707	NA
S_10	USP29	19:57642592:A,T	nonsynonymous SNV	p.K850M	0	0	0	0	0,150943396	NA
S_10	USP42	7:6193802:G,C	nonsynonymous SNV	p.V873L	0	0	0	0	0,180392157	NA
S_10	USP6NL	10:11639630:T,G	nonsynonymous SNV	p.N2H	0	0	0	0	0,43902439	NA
S_10	UTP18	17:49354641:T,G	nonsynonymous SNV	p.L330V	0	0	0	0	0,28125	NA
S_10	VAMP8	2:85806261:C,A	nonsynonymous SNV	p.R45S	0	0	0	0	0,212765957	NA
S_10	WARS2	1:119584968:T,G	nonsynonymous SNV	p.K145T	0	0	0	0	0,5	NA
S_10	WDFY3	4:85729569:G,C	nonsynonymous SNV	p.R783G	0	0	0	0	0,193548387	NA
S_10	WDR59	16:75018885:C,G	nonsynonymous SNV	p.V11L	0	0	0	0	0,147766323	NA
S_10	WDR66	12:122359581:A,C	nonsynonymous SNV	p.I124L	0	0	0	0	0,279661017	NA

S_10	WDR72	15:53998144:T,G	nonsynonymous SNV	p.K361T		0	0	0	0	0,194444444	NA
S_10	WDR72	15:54003568:T,G	nonsynonymous SNV	p.E274D		0	0	0	0	0,156862745	NA
S_10	WNK4	17:40947708:C,A	nonsynonymous SNV	p.P1030T		0	0	0	0	0,30075188	NA
S_10	ZBED2	3:111312729:C,G	nonsynonymous SNV	p.R107T		0	0	0	0	0,374172185	NA
S_10	ZBTB4	17:7369604:A,G	nonsynonymous SNV	p.S173P		0	0	0	0,00877193	0,413333333	5,07E-16
S_10	ZBTB8B	1:32946614:G,C	nonsynonymous SNV	p.E372Q		0	0	0	0	0,358974359	NA
S_10	ZCCHC17	1:31836892:A,C	nonsynonymous SNV	p.K215T		0	0	0	0	0,275	NA
S_10	ZFHX4	8:77766148:C,T	stopgain	p.Q2331X		0	0	0	0	0,174496644	NA
S_10	ZFP62	5:180278124:C,T	nonsynonymous SNV	p.G124E		0	0	0	0	0,285714286	NA
S_10	ZKSCAN7	3:44612227:A,C	nonsynonymous SNV	p.Q542P		0	0	0	0	0,5	NA
S_10	ZNF12	7:6737000:C,T	nonsynonymous SNV	p.E70K		0	0	0	0	0,186046512	NA
S_10	ZNF175	19:52084659:G,A	nonsynonymous SNV	p.E30K		0	0	0	0	0,267857143	NA
S_10	ZNF207	17:30685568:G,A	nonsynonymous SNV	p.R72K		0	0	0	0,009708738	0,357142857	8,66E-06
S_10	ZNF213	16:3187511:A,C	nonsynonymous SNV	p.E77A		0	0	0	0	0,311111111	NA
S_10	ZNF331	19:54074972:T,G	nonsynonymous SNV	p.L42V		0	0	0	0	0,216216216	NA
S_10	ZNF347	19:53645601:T,G	nonsynonymous SNV	p.E161D		0	0	0	0	0,232142857	NA
S_10	ZNF414	19:8576606:A,C	nonsynonymous SNV	p.L257V		0	0	0	0	0,35483871	NA
S_10	ZNF512B	20:62593342:A,G	nonsynonymous SNV	p.Y761H		0	0	0	0	0,260504202	NA
S_10	ZNF70	22:24086117:G,T	stopgain	p.S404X		0	0	0	0	0,309677419	NA
S_10	ZSCAN30	18:32843956:T,G	nonsynonymous SNV	p.T121P		0	0	0	0	0,26848249	NA
S_11	ZNF222	19:44531205:A,G	nonsynonymous SNV	p.I25V	rs1063264	0,0014	0	0	0	0,260869565	NA
S_11	KCTD17	22:37449212:G,A	nonsynonymous SNV	p.G95S		8,14E-06	0	0	0	0,348214286	NA
S_11	PTPRS	19:5231539:G,A	nonsynonymous SNV	p.T633M		8,15E-06	0	0	0,095238095	0,393442623	0,00010032
S_11	FLG2	1:152324407:C,G	nonsynonymous SNV	p.G1952A		1,63E-05	0	0	0,005555556	0,114942529	0,0006732
S_11	ACHE	7:100491715:G,A	nonsynonymous SNV	p.R47W		0	0	0	0,036585366	0,383333333	1,72E-08
S_11	ANK3	10:61956359:C,G	nonsynonymous SNV	p.A555P		0	0	0	0	0,341772152	NA
S_11	API5	11:43345112:A,G	nonsynonymous SNV	p.T172A		0	0	0	0,101010101	0,421052632	0,000571
S_11	ASXL2	2:25967109:T,A	nonsynonymous SNV	p.Q699H		0	0	0	0	0,079787234	NA
S_11	BCL11B	14:99641369:C,T	nonsynonymous SNV	p.V530M		0	0	0	0,01025641	0,079734219	0,0021176
S_11	CCND2	12:4398076:C,A	nonsynonymous SNV	p.L214I		0	0	0	0	0,32	NA
S_11	CD5	11:60886805:A,C	nonsynonymous SNV	p.E188A		0	0	0	0	0,391089109	NA
S_11	EFCAB5	17:28400204:A,G	nonsynonymous SNV	p.N839S		0	0	0	0	0,4375	NA
S_11	GLI1	12:57865415:-,A	frameshift insertion	p.Y836fs		0	0	0	NA	0,3125	NA
S_11	IPO7	11:9450605:G,-	frameshift deletion	p.E485fs		0	0	0	NA	0,5	NA
S_11	KAT6B	10:76735849:-,CT	frameshift insertion	p.A585fs		0	0	0	NA	0,320754717	NA
S_11	KAT6B	10:76735852:-,C	frameshift insertion	p.H586fs		0	0	0	NA	0,314814815	NA
S_11	LHX5	12:113905171:A,C	nonsynonymous SNV	p.L244R		0	0	0	0,128571429	0,384126984	1,20E-07
S_11	MIA3	1:222825337:TCT,-	nonframeshift deletion	p.188_188del		0	0	0	NA	0,5625	NA
S_11	NDUFA4	7:10978434:CC,-	frameshift deletion	p.C44fs		0	0	0	NA	0,333333333	NA
S_11	NDUFA4	7:10978441:T,-	frameshift deletion	p.D42fs		0	0	0	NA	0,333333333	NA
S_11	THBS3	1:155168285:-,AAG	nonframeshift insertion	p.D543delinsDF		0	0	0	NA	0,333333333	NA
S_11	ZNF182	X:47835908:CT,-	frameshift deletion	p.K507fs		0	0	0	NA	0,333333333	NA
S_11	ZNF623	8:144732519:-,G	frameshift insertion	p.G159fs		0	0	0	NA	0,333333333	NA
S_2	MUC7	4:71347060:A,C	nonsynonymous SNV	p.Q200P	rs74904873	0	0	0,001	NA	0,091666667	NA
S_2	COX5A	15:75230282:G,C	nonsynonymous SNV	p.S25C	rs200367305	0,000634	0	0,003	NA	0,275862069	NA
S_2	MAGEC1	X:140994346:C,G	nonsynonymous SNV	p.L386V	rs60520741	0,00213	0	0,003	NA	0,068181818	NA
S_2	CYP4F12	19:15784370:C,T	nonsynonymous SNV	p.L11F	rs181648518	0,00391	0,005466	0,007	NA	0,138211382	NA
S_2	ANO3	11:26353862:C,G	nonsynonymous SNV	p.Q14E	rs142688782	2,44E-05	0,000116	0	NA	0,208333333	NA

S_2	ZNF721	4:437602:A,C	nonsynonymous SNV	p.D218E	rs368280658	0	0,000353	0	NA	0,062992126	NA	
S_2	PARM1	4:75937991:G,C	nonsynonymous SNV	p.V134L	rs200120426	8,15E-06	0	0	NA	0,176744186	NA	
S_2	SERPINA10	14:94756791:T,C	nonsynonymous SNV	p.E47G		8,13E-06	0	0	NA	0,248407643	NA	not tested
S_2	RANBP3	19:5932489:G,A	nonsynonymous SNV	p.P112L		8,17E-06	0	0	NA	0,218340611	NA	
S_2	ANO1	11:70028645:G,A	nonsynonymous SNV	p.R814H		1,63E-05	0	0	NA	0,188442211	NA	
S_2	ZNF679	7:63727074:A,G	nonsynonymous SNV	p.K355E		2,45E-05	0	0	NA	0,117647059	NA	
S_2	ZNF732	4:265671:G,C	nonsynonymous SNV	p.N324K		4,89E-05	0	0	NA	0,115384615	NA	
S_2	GZMM	19:548947:G,A	nonsynonymous SNV	p.R86Q		4,92E-05	0	0	NA	0,214285714	NA	
S_2	CTCFL	20:56075449:G,A	nonsynonymous SNV	p.R358C		7,68E-05	0	0	NA	0,060150376	NA	
S_2	ATXN1	6:16327921:C,A	nonsynonymous SNV	p.Q207H	rs201030692	0,00317	0	0	NA	0,277227723	NA	
S_2	ABHD5	3:43759187:G,C	nonsynonymous SNV	p.M266I		0	0	0	NA	0,365853659	NA	
S_2	ADAMTS8	11:130275547:C,G	nonsynonymous SNV	p.C859S		0	0	0	NA	0,116071429	NA	
S_2	AP1B1	22:29727846:G,C	nonsynonymous SNV	p.T763R		0	0	0	NA	0,236486486	NA	
S_2	ASXL1	20:31019232:T,A	stopgain	p.L276X		0	0	0	NA	0,162650602	NA	
S_2	ATP2A3	17:3844486:C,G	nonsynonymous SNV	p.D627H		0	0	0	NA	0,264851485	NA	
S_2	ATP2B4	1:203652380:A,G	nonsynonymous SNV	p.E16G		0	0	0	NA	0,21686747	NA	
S_2	ATXN1	6:16327907:TGA,-	nonframeshift deletion	p.211_212del		0	0	0	NA	0,41322314	NA	
S_2	BCAN	1:156617401:G,A	nonsynonymous SNV	p.E190K		0	0	0	NA	0,182795699	NA	
S_2	BSN	3:49701256:G,T	nonsynonymous SNV	p.A3849S		0	0	0	NA	0,180952381	NA	
S_2	DIXDC1	11:111866226:G,A	unknown	NA		0	0	0	NA	0,196721311	NA	
S_2	DNAH17	17:76450605:C,A	nonsynonymous SNV	p.W3451C		0	0	0	NA	0,145374449	NA	
S_2	ELP4	11:31703575:T,C	nonsynonymous SNV	p.Y462H		0	0	0	NA	0,064	NA	
S_2	EPS8	12:15774256:G,A	nonsynonymous SNV	p.H822Y		0	0	0	NA	0,24137931	NA	
S_2	FUBP1	1:78435647:T,G	nonsynonymous SNV	p.Y58S		0	0	0	NA	0,25	NA	
S_2	FXYD6,FXYD6-FXYD2	11:117711043:T,C	nonsynonymous SNV	p.T84A		0	0	0	NA	0,197530864	NA	
S_2	HGC6.3	6:168377176:G,T	nonsynonymous SNV	p.H53N		0	0	0	NA	0,161290323	NA	
S_2	HMCN1	1:185986194:C,G	nonsynonymous SNV	p.P1764R		0	0	0	NA	0,259259259	NA	
S_2	IGFN1	1:201179785:A,G	nonsynonymous SNV	p.N1922D		0	0	0	NA	0,090909091	NA	
S_2	IGFN1	1:201179788:G,A	nonsynonymous SNV	p.E1923K		0	0	0	NA	0,083333333	NA	
S_2	IGFN1	1:201179821:G,A	nonsynonymous SNV	p.E1934K	rs202024614	0	0	0	NA	0,133928571	NA	
S_2	IL27RA	19:14162448:G,T	nonsynonymous SNV	p.G520C		0	0	0	NA	0,225806452	NA	
S_2	LARGE	22:33670611:C,A	NA	NA		0	0	0	NA	0,2	NA	
S_2	LATS1	6:150005620:G,A	nonsynonymous SNV	p.S202F		0	0	0	NA	0,357142857	NA	
S_2	MAD2L1BP	6:43604288:A,T	nonsynonymous SNV	p.T73S		0	0	0	NA	0,161290323	NA	
S_2	MAGEB18	X:26157376:A,C	nonsynonymous SNV	p.S92R		0	0	0	NA	0,2	NA	
S_2	MAP4K1	19:39079857:T,C	nonsynonymous SNV	p.N819D		0	0	0	NA	0,091954023	NA	
S_2	MUC16	19:9073963:C,G	nonsynonymous SNV	p.D4495H		0	0	0	NA	0,162162162	NA	
S_2	NDUFA4	7:10978434:CC,-	frameshift deletion	p.C44fs		0	0	0	NA	0,368421053	NA	
S_2	NDUFA4	7:10978441:T,-	frameshift deletion	p.D42fs		0	0	0	NA	0,333333333	NA	
S_2	NLGN1	3:173997027:C,G	nonsynonymous SNV	p.D412E		0	0	0	NA	0,088235294	NA	
S_2	NLRC4	2:32476398:G,C	nonsynonymous SNV	p.L179V		0	0	0	NA	0,264957265	NA	
S_2	NUDT7	16:77769348:T,C	nonsynonymous SNV	p.S83P		0	0	0	NA	0,078125	NA	
S_2	OR1D2	17:2996204:C,T	stopgain	p.W29X		0	0	0	NA	0,397058824	NA	
S_2	PCCB	3:136035809:G,C	nonsynonymous SNV	p.E331D		0	0	0	NA	0,170731707	NA	
S_2	PLEKHD1	14:69989022:T,C	nonsynonymous SNV	p.V193A		0	0	0	NA	0,32	NA	
S_2	PML	15:74335364:C,T	nonsynonymous SNV	p.S534F		0	0	0	NA	0,307692308	NA	
S_2	PPFIA3	19:49652903:C,T	nonsynonymous SNV	p.P1152S		0	0	0	NA	0,225165563	NA	

S_2	PRR12	19:50099492:G,C	nonsynonymous SNV	p.E634Q		0	0	0	NA	0,247787611	NA	
S_2	PRR21	2:240981459:A,G	nonsynonymous SNV	p.M314T	rs147134334	0	0	0	NA	0,090909091	NA	
S_2	PSMD2	3:184017130:G,A	nonsynonymous SNV	p.E26K		0	0	0	NA	0,13253012	NA	
S_2	PTH1R	3:46944820:A,G	nonsynonymous SNV	p.K486E		0	0	0	NA	0,303797468	NA	
S_2	RPH3AL	17:96955:C,A	nonsynonymous SNV	p.R158L		0	0	0	NA	0,176470588	NA	
S_2	SHANK2	11:70331948:C,G	nonsynonymous SNV	p.D896H		0	0	0	NA	0,182692308	NA	
S_2	SLC25A23	19:6444179:G,C	nonsynonymous SNV	p.T402S		0	0	0	NA	0,196428571	NA	
S_2	SOCS7	17:36508676:G,C	nonsynonymous SNV	p.Q183H		0	0	0	NA	0,288	NA	
S_2	SPRR3	1:152976000:G,C	nonsynonymous SNV	p.Q168H		0	0	0	NA	0,189189189	NA	
S_2	SYNCRIP	6:86324694:C,A	nonsynonymous SNV	p.R516L		0	0	0	NA	0,28042328	NA	
S_2	TCF20	22:42610464:G,C	nonsynonymous SNV	p.A283G		0	0	0	NA	0,231578947	NA	
S_2	TENM4	11:78387259:C,A	stopgain	p.E1812X		0	0	0	NA	0,152542373	NA	
S_2	TMEM132E	17:32964991:G,C	nonsynonymous SNV	p.G899R		0	0	0	NA	0,290909091	NA	
S_2	TNS1	2:218750496:G,A	nonsynonymous SNV	p.P238S		0	0	0	NA	0,176470588	NA	
S_2	TPR	1:186313623:C,T	nonsynonymous SNV	p.E1101K		0	0	0	NA	0,220640569	NA	
S_2	TRPM2	21:45786701:G,C	nonsynonymous SNV	p.G163A		0	0	0	NA	0,182857143	NA	
S_2	TTN	2:179639045:A,G	nonsynonymous SNV	p.S2270P		0	0	0	NA	0,164556962	NA	
S_2	TUBA8	22:18609718:C,T	nonsynonymous SNV	p.P259S		0	0	0	NA	0,166219839	NA	
S_2	USP2	11:119229501:C,T	nonsynonymous SNV	p.E164K		0	0	0	NA	0,127118644	NA	
S_2	USPL1	13:31232690:A,C	nonsynonymous SNV	p.S826R		0	0	0	NA	0,244897959	NA	
S_2	ZNF208	19:22156484:G,T	nonsynonymous SNV	p.T451K		0	0	0	NA	0,103896104	NA	
S_2	ZNF254	19:24309659:T,G	nonsynonymous SNV	p.I201R		0	0	0	NA	0,1	NA	
S_2	ZNF292	6:87970767:G,A	nonsynonymous SNV	p.E2474K		0	0	0	NA	0,170212766	NA	
S_2	ZNF486	19:20308211:C,T	nonsynonymous SNV	p.T231I		0	0	0	NA	0,098591549	NA	
S_2	ZNF695	1:247131049:G,C	nonsynonymous SNV	p.L146V		0	0	0	NA	0,127659574	NA	
S_2	ZNF732	4:265271:C,T	nonsynonymous SNV	p.A458T		0	0	0	NA	0,104166667	NA	
S_2	ZNF776	19:58265844:A,G	nonsynonymous SNV	p.Q449R	rs201940964	0	0	0	NA	0,104761905	NA	
S_2	ZNF99	19:22940265:A,G	nonsynonymous SNV	p.S816P		0	0	0	NA	0,153846154	NA	
S_2	ZSCAN23	6:28403352:AAA,-	nonframeshift deletion	p.147_147del		0	0	0	NA	0,5	NA	
S_3	ECSIT	19:11618651:C,T	nonsynonymous SNV	p.D57N	rs373495105	1,63E-05	0	0	0,23566879	0,475806452	0,0011556	
S_3	ZNF774	15:90903656:G,A	nonsynonymous SNV	p.R198H	rs201354012	0,000708	0	0	0,198113208	0,385620915	0,00336	
S_3	SPATA5	4:124177318:G,A	nonsynonymous SNV	p.A830T		8,13E-06	0	0	0,536423841	1	9,82E-16	
S_3	ABCA4	1:94486866:C,T	nonsynonymous SNV	p.E1650K		1,63E-05	0	0	0,686131387	1	5,87E-11	
S_3	CASR	3:122003779:C,T	nonsynonymous SNV	p.T993M		1,63E-05	0	0	0,139013453	0,266666667	0,00024976	
S_3	FAM13A	4:89950679:C,T	nonsynonymous SNV	p.R50Q		3,25E-05	0	0	0	0,368421053	NA	
S_3	NDST1	5:149900959:C,T	nonsynonymous SNV	p.S48L		4,88E-05	0	0	0,002463054	0,222988506	1,82E-27	
S_3	C1QB	1:22986016:G,A	nonsynonymous SNV	p.D23N		0,00013	0	0	0,447058824	0,776119403	0,0017056	validated
S_3	ADAM10	15:58971462:G,T	nonsynonymous SNV	p.S115R		0	0	0	0	0,294117647	NA	
S_3	ADORA1	1:203134721:A,T	nonsynonymous SNV	p.Y225F		0	0	0	0,001964637	0,236051502	1,20E-35	
S_3	AKAP12	6:151673231:A,T	nonsynonymous SNV	p.E1137D		0	0	0	0	0,186119874	NA	
S_3	ANKRD30B	18:14778069:T,G	nonsynonymous SNV	p.I472R		0	0	0	0,333333333	0,941176471	0,0005137	
S_3	ARX	X:25025537:C,T	nonsynonymous SNV	p.R380Q		0	0	0	0	0,151898734	NA	
S_3	CCAR2	8:22472489:A,G	nonsynonymous SNV	p.I394V		0	0	0	0,47826087	1	0,0013727	
S_3	CCDC102B	18:66504300:-,A	frameshift insertion	p.A100fs		0	0	0	NA	0,347619048	NA	
S_3	CCL2	17:32582419:C,G	nonsynonymous SNV	p.F17L		0	0	0	0,24609375	0,5	3,34E-06	
S_3	COL4A2	13:111145562:T,G	nonsynonymous SNV	p.F1189L		0	0	0	0,558823529	0,990825688	4,21E-17	
S_3	CUBN	10:17171683:G,A	nonsynonymous SNV	p.L28F		0	0	0	0,468899522	0,669064748	0,008035584	
S_3	CYP2S1	19:41707262:T,C	nonsynonymous SNV	p.Y321H		0	0	0	0,633802817	1	2,66E-08	

S_3	DBF4B	17:42814213:G,T	nonsynonymous SNV	p.A216S		0	0	0	0,216374269	0,438095238	0,0045619	
S_3	DRD4	11:637467:G,A	nonsynonymous SNV	p.V55M		0	0	0	0,229508197	0,617647059	0,009799876	
S_3	FAM189B	1:155220419:G,T	nonsynonymous SNV	p.F290L		0	0	0	0	0,183574879	NA	
S_3	FASTKD5	20:3128063:C,G	nonsynonymous SNV	p.D552H		0	0	0	0,158415842	0,300395257	0,0024378	
S_3	FBXO48	2:68691364:G,C	nonsynonymous SNV	p.L149V		0	0	0	0	0,222641509	NA	
S_3	FBXO48	2:68692187:G,A	stopgain	p.Q31X		0	0	0	0	0,19047619	NA	
S_3	FGD1	X:54521717:G,A	nonsynonymous SNV	p.S50L		0	0	0	0	0,295081967	NA	
S_3	GATA3	10:8111542:-,C	frameshift insertion	p.L344fs		0	0	0	0,396226415	0,638709677	3,44E-05	
S_3	GATSL3	22:30682937:C,A	nonsynonymous SNV	p.G169V		0	0	0	0,416	0,72	0,00022686	
S_3	HOXA2	7:27142068:G,A	nonsynonymous SNV	p.L18F		0	0	0	0	0,352459016	NA	
S_3	HTR5A	7:154862905:T,G	nonsynonymous SNV	p.V99G		0	0	0	0	0,060606061	NA	
S_3	IL26	12:68595671:T,A	nonsynonymous SNV	p.D157V		0	0	0	0	0,1875	NA	
S_3	LRP1	12:57574470:G,A	nonsynonymous SNV	p.D1803N		0	0	0	0	0,246666667	NA	
S_3	MCF2L	13:113736831:G,C	nonsynonymous SNV	p.E656Q		0	0	0	0,48	1	1,32E-05	
S_3	OCA2	15:28000560:C,A	nonsynonymous SNV	p.A807S		0	0	0	0	0,295681063	NA	
S_3	OCSTAMP	20:45169923:C,G	nonsynonymous SNV	p.R564T		0	0	0	0	0,227722772	NA	
S_3	OR5B3	11:58170693:A,T	nonsynonymous SNV	p.L64M		0	0	0	0	0,431693989	NA	
S_3	PHKA1	X:71840622:C,T	nonsynonymous SNV	p.C697Y		0	0	0	0	0,133333333	NA	
S_3	PML	15:74290729:-,A	frameshift insertion	p.Q172fs		0	0	0	0,402489627	0,655172414	7,49E-12	
S_3	PTDSS1	8:97342501:C,T	stopgain	p.R266X		0	0	0	0	0,204545455	NA	
S_3	RLF	1:40704799:T,-	frameshift deletion	p.D1475fs		0	0	0	NA	0,342307692	NA	
S_3	SAMD9	7:92733987:G,T	nonsynonymous SNV	p.T475K		0	0	0	0,484375	1	3,38E-21	
S_3	SFRP5	10:99529431:C,A	nonsynonymous SNV	p.D201Y		0	0	0	0,388888889	0,703125	0,009799876	
S_3	SWAP70	11:9685809:C,G	nonsynonymous SNV	p.S28C		0	0	0	0,585714286	1	2,52E-08	
S_3	ZNF665	19:53669237:T,G	nonsynonymous SNV	p.K169T		0	0	0	0	0,402985075	NA	
S_4	ZNF202	11:123601200:G,A	nonsynonymous SNV	p.R133W	rs369977165	8,29E-06	0,000116	0	NA	0,825	NA	
S_4	AEBP2	12:19592829:GGCGGCGGAGGC,-	nonframeshift deletion	p.66_69del	rs558021601	0,00182	0,006592	0	NA	0,52	NA	
S_4	DDIT4	10:74034581:C,G	nonsynonymous SNV	p.R112G	rs147217433	1,63E-05	0	0	NA	0,087248322	NA	
S_4	TCHHL1	1:152059329:C,T	nonsynonymous SNV	p.E277K	rs114173361	0,000407	0	0	NA	0,242753623	NA	
S_4	F5	1:169510399:G,C	nonsynonymous SNV	p.T1310R		8,13E-06	0	0	NA	0,057692308	NA	not tested
S_4	PRG4	1:186277277:G,A	nonsynonymous SNV	p.G675E	rs113308576	8,13E-06	0	0	NA	0,097444089	NA	
S_4	ZNF728	19:23159061:C,T	nonsynonymous SNV	p.V360I		8,15E-06	0	0	NA	0,2	NA	
S_4	NR1H2	19:50885339:C,T	nonsynonymous SNV	p.R285W		9,30E-06	0	0	NA	0,268817204	NA	
S_4	ZNF679	7:63727143:G,A	nonsynonymous SNV	p.E378K		1,63E-05	0	0	NA	0,107142857	NA	
S_4	ZNF256	19:58452611:T,C	nonsynonymous SNV	p.N522S		4,07E-05	0	0	NA	0,054421769	NA	
S_4	IGFN1	1:201180101:G,A	nonsynonymous SNV	p.R2027Q	rs527808016	4,91E-05	0	0	NA	0,111607143	NA	
S_4	ZNF90	19:20230036:G,T	nonsynonymous SNV	p.S558I	rs552166239	9,78E-05	0	0	NA	0,153846154	NA	
S_4	OR2L8	1:248112794:G,C	nonsynonymous SNV	p.G212A	rs200574966	0,00013	0	0	NA	0,166666667	NA	
S_4	IGFN1	1:201179829:G,A	nonsynonymous SNV	p.M1936I		0,000144	0	0	NA	0,053003534	NA	
S_4	IGFN1	1:201180551:G,A	nonsynonymous SNV	p.G2177D	rs545602248	0,00211	0	0	NA	0,121281465	NA	
S_4	CD46	1:207930511:C,T	nonsynonymous SNV	p.H84Y		0	0	0	NA	0,361445783	NA	not tested
S_4	ACVR1C	2:158443823:G,C	nonsynonymous SNV	p.I10M		0	0	0	NA	0,432432432	NA	
S_4	ATP5G2	12:54069856:G,A	nonsynonymous SNV	p.L41F		0	0	0	NA	0,312977099	NA	
S_4	B3GNT6	11:76750760:C,G	unknown	NA		0	0	0	NA	0,062385321	NA	
S_4	BARD1	2:215674175:G,C	nonsynonymous SNV	p.A40G		0	0	0	NA	0,641509434	NA	
S_4	C10orf71	10:50533864:G,C	nonsynonymous SNV	p.E1092Q		0	0	0	NA	0,099447514	NA	
S_4	C16orf91	16:1470348:C,A	stopgain	p.E100X		0	0	0	NA	0,250554324	NA	
S_4	CACNA1A	19:13563783:C,T	nonsynonymous SNV	p.G149E		0	0	0	NA	0,916666667	NA	

S_4	CASS4	20:55027691:G,A	nonsynonymous SNV	p.E433K		0	0	0	NA	0,076923077	NA
S_4	CCDC92	12:124421792:G,A	nonsynonymous SNV	p.S270F		0	0	0	NA	0,059382423	NA
S_4	CHST5	16:75563311:C,T	stopgain	p.W324X		0	0	0	NA	0,104166667	NA
S_4	CNGA3	2:99012756:G,C	nonsynonymous SNV	p.D357H		0	0	0	NA	0,333333333	NA
S_4	COL6A2	21:47545414:T,A	nonsynonymous SNV	p.F618I		0	0	0	NA	0,095398429	NA
S_4	COL6A2	21:47545941:C,T	nonsynonymous SNV	p.R738W		0	0	0	NA	0,118032787	NA
S_4	CUX2	12:111747892:G,A	nonsynonymous SNV	p.E436K		0	0	0	NA	0,385964912	NA
S_4	DDX49	19:19038978:G,T	nonsynonymous SNV	p.K435N		0	0	0	NA	0,111675127	NA
S_4	DYNC1I1	7:95739378:A,G	nonsynonymous SNV	p.T602A		0	0	0	NA	0,082644628	NA
S_4	EIF2B3	1:45316661:C,T	nonsynonymous SNV	p.E441K		0	0	0	NA	0,763157895	NA
S_4	EPDR1	7:37988520:G,A	nonsynonymous SNV	p.M55I		0	0	0	NA	0,227272727	NA
S_4	FAM178A	10:102683828:G,A	nonsynonymous SNV	p.R357K		0	0	0	NA	0,258064516	NA
S_4	FLT3	13:28602317:G,C	stopgain	p.S684X		0	0	0	NA	0,666666667	NA
S_4	FOXB1	15:60297378:C,G	nonsynonymous SNV	p.I72M		0	0	0	NA	0,465116279	NA
S_4	GBGT1	9:136029213:G,C	nonsynonymous SNV	p.F248L		0	0	0	NA	0,394366197	NA
S_4	IGFN1	1:201179782:G,A	nonsynonymous SNV	p.V1921M		0	0	0	NA	0,067164179	NA
S_4	IGFN1	1:201179785:A,G	nonsynonymous SNV	p.N1922D		0	0	0	NA	0,0625	NA
S_4	IGFN1	1:201179788:G,A	nonsynonymous SNV	p.E1923K		0	0	0	NA	0,08125	NA
S_4	JAG1	20:10632817:G,A	nonsynonymous SNV	p.S323F		0	0	0	NA	0,565217391	NA
S_4	KCNJ9	1:160054511:G,A	nonsynonymous SNV	p.G231S		0	0	0	NA	0,191489362	NA
S_4	KIAA0408	6:127767648:C,A	stopgain	p.E606X		0	0	0	NA	0,188888889	NA
S_4	LONP1	19:5707106:C,G	nonsynonymous SNV	p.E175Q		0	0	0	NA	0,178571429	NA
S_4	MAST4	5:66300825:G,C	nonsynonymous SNV	p.D2H		0	0	0	NA	0,376146789	NA
S_4	MIA3	1:222825337:TCT,-	nonframeshift deletion	p.188_188del		0	0	0	NA	0,6	NA
S_4	MPZ	1:161275908:C,T	nonsynonymous SNV	p.R212H		0	0	0	NA	0,303921569	NA
S_4	MRPS31	13:41333162:T,A	nonsynonymous SNV	p.K174M		0	0	0	NA	0,368421053	NA
S_4	OXSR1	3:38240282:C,T	nonsynonymous SNV	p.S121F		0	0	0	NA	0,346153846	NA
S_4	PYGM	11:64514234:G,A	nonsynonymous SNV	p.S721F		0	0	0	NA	0,064308682	NA
S_4	RALGAPA2	20:20693113:C,T	nonsynonymous SNV	p.R4Q		0	0	0	NA	0,083333333	NA
S_4	RGAG1	X:109697425:C,G	nonsynonymous SNV	p.L1194V		0	0	0	NA	0,193548387	NA
S_4	RHOBTB3	5:95088025:G,T	nonsynonymous SNV	p.G218V		0	0	0	NA	0,340909091	NA
S_4	RYR1	19:39057581:G,A	nonsynonymous SNV	p.E4485K		0	0	0	NA	0,257731959	NA
S_4	SEMA4G	10:102732906:T,G	nonsynonymous SNV	p.F49V		0	0	0	NA	0,172661871	NA
S_4	SOGA1	20:35457461:C,T	nonsynonymous SNV	p.R356K		0	0	0	NA	0,09009009	NA
S_4	TLL2	10:98155742:G,C	nonsynonymous SNV	p.Q474E		0	0	0	NA	0,5625	NA
S_4	TMEM27	X:15682856:C,T	nonsynonymous SNV	p.E15K		0	0	0	NA	0,11627907	NA
S_4	TMEM39B	1:32542878:C,G	nonsynonymous SNV	p.F183L		0	0	0	NA	0,301507538	NA
S_4	TPH2	12:72338203:C,G	nonsynonymous SNV	p.Q129E		0	0	0	NA	0,103448276	NA
S_4	TRABD	22:50632792:C,T	nonsynonymous SNV	p.L46F		0	0	0	NA	0,128205128	NA
S_4	ZBTB45	19:59028713:G,A	nonsynonymous SNV	p.R110C		0	0	0	NA	0,177777778	NA
S_4	ZFHX2	14:23998974:G,T	stopgain	p.S960X		0	0	0	NA	0,4375	NA
S_4	ZNF429	19:21720557:A,G	nonsynonymous SNV	p.K568E		0	0	0	NA	0,089552239	NA
S_4	ZNF43	19:21990645:G,T	nonsynonymous SNV	p.Q667K		0	0	0	NA	0,094339623	NA
S_4	ZNF676	19:22363101:G,C	nonsynonymous SNV	p.A473G		0	0	0	NA	0,147058824	NA
S_4	ZNF726	19:24116263:A,G	nonsynonymous SNV	p.R449G		0	0	0	NA	0,104166667	NA
S_4	ZNF727	7:63538088:G,A	nonsynonymous SNV	p.V221I		0	0	0	NA	0,090909091	NA
S_4	ZNF728	19:23159060:A,C	nonsynonymous SNV	p.V360G		0	0	0	NA	0,11627907	NA
S_4	ZNF761	19:53958879:A,G	nonsynonymous SNV	p.H373R	rs543658141	0	0	0	NA	0,128205128	NA

S_4	ZNF844	19:12187275:G,C	nonsynonymous SNV	p.R447P		0	0	0	NA	0,208333333	NA	
S_5	VWF	12:6172182:G,A	nonsynonymous SNV	p.R491C	rs372904370	0	0,000116	0	0,020833333	0,310344828	0,004108275	not tested
S_5	ZFHX4	8:77766341:C,T	nonsynonymous SNV	p.P2395L		8,17E-06	0	0	0	0,115577889	NA	
S_5	CCDC80	3:112357953:C,T	nonsynonymous SNV	p.R267H		1,63E-05	0	0	0,014184397	0,129032258	0,0002676	
S_5	FLG2	1:152327047:C,T	nonsynonymous SNV	p.R1072H		5,69E-05	0	0	0,011538462	0,102272727	0,003672163	
S_5	CPEB2	4:15004815:A,G	nonsynonymous SNV	p.Q173R		0	0	0	0,021834061	0,145631068	1,51E-05	
S_5	CTU1	19:51607664:C,T	nonsynonymous SNV	p.A55T		0	0	0	0	0,214285714	NA	
S_5	GBE1	3:81539581:G,T	nonsynonymous SNV	p.Q696K		0	0	0	0,017857143	0,25	0,006841832	
S_5	GREM2	1:240656492:C,T	nonsynonymous SNV	p.R95H		0	0	0	0	0,116883117	NA	
S_5	HSPA12B	20:3730459:G,A	nonsynonymous SNV	p.E333K		0	0	0	0	0,072289157	NA	
S_5	INPP4A	2:99160375:C,T	nonsynonymous SNV	p.P285L		0	0	0	0	0,136363636	NA	
S_5	INPP4A	2:99160442:G,T	nonsynonymous SNV	p.E307D		0	0	0	0	0,133333333	NA	
S_5	KBTBD6	13:41705959:C,T	nonsynonymous SNV	p.S230N		0	0	0	0	0,347826087	NA	
S_5	KLK6	19:51462546:A,C	nonsynonymous SNV	p.C96W		0	0	0	0	0,32	NA	
S_5	LGMN	14:93180172:C,A	nonsynonymous SNV	p.R180L		0	0	0	0	0,5	NA	
S_5	LIG1	19:48638998:T,A	nonsynonymous SNV	p.T420S		0	0	0	0	0,224489796	NA	
S_5	LIG1	19:48639019:C,A	nonsynonymous SNV	p.V413L		0	0	0	0	0,161290323	NA	
S_5	MED15	22:20918914:T,A	nonsynonymous SNV	p.L139H		0	0	0	0	0,088888889	NA	
S_5	ODF1	8:103573033:A,G	nonsynonymous SNV	p.N225S	rs62523272	0	0	0	0	0,212765957	NA	
S_5	PKP2	12:32996129:C,A	nonsynonymous SNV	p.Q499H		0	0	0	0	0,157894737	NA	
S_5	SPEG	2:220312856:G,A	nonsynonymous SNV	p.A326T		0	0	0	0	0,060810811	NA	
S_5	TMEM132B	12:125834209:T,A	stopgain	p.Y88X		0	0	0	0	0,125	NA	
S_5	TOR4A	9:140173289:G,A	nonsynonymous SNV	p.G50R		0	0	0	0	0,116129032	NA	
S_5	TTC3	21:38459693:G,A	nonsynonymous SNV	p.D46N		0	0	0	0	0,095588235	NA	
S_5	ZNF225	19:44635770:C,T	nonsynonymous SNV	p.R335C		0	0	0	0,008097166	0,12	0,00372057	
S_5	ZNF347	19:53644267:A,G	nonsynonymous SNV	p.V606A		0	0	0	0	0,184210526	NA	
S_5	ZNF695	1:247151018:T,G	nonsynonymous SNV	p.T267P		0	0	0	0	0,152941176	NA	
S_5	ZNF695	1:247151024:T,C	nonsynonymous SNV	p.K265E		0	0	0	0	0,153846154	NA	
S_5	ZNF776	19:58265844:A,G	nonsynonymous SNV	p.Q449R	rs201940964	0	0	0	0,005319149	0,139534884	7,62E-06	
S_6	TCHH	1:152082544:T,C	nonsynonymous SNV	p.Y1050C	rs540863755	8,16E-06	0	0,001	0	0,150485437	NA	
S_6	ZNF728	19:23158534:T,G	nonsynonymous SNV	p.Q535H	rs186425609	1,64E-05	0	0,001	0	0,144578313	NA	
S_6	BCAS4	20:49492562:G,A	nonsynonymous SNV	p.R173H	rs7273412	0,00546	0	0,001	0	0,189873418	NA	
S_6	FLG2	1:152324371:G,T	nonsynonymous SNV	p.P1964H	rs140669858	0,000895	0	0,002	0,012987013	0,25	2,90E-05	
S_6	ZNF93	19:20026199:T,A	nonsynonymous SNV	p.S38R	rs150187903	1,63E-05	0,000116	0	0	0,112244898	NA	
S_6	LPCAT1	5:1494983:C,T	nonsynonymous SNV	p.A109T	rs140317722	3,26E-05	0,000116	0	0	0,210084034	NA	
S_6	HRNR	1:152191944:T,C	nonsynonymous SNV	p.S721G	rs77232056	0,00325	0,000233	0	0	0,125	NA	
S_6	ZNF737	19:20736568:C,T	nonsynonymous SNV	p.R26Q	rs368441798	0,000106	0,000314	0	0	0,094339623	NA	
S_6	ADAMTS14	10:72511968:C,T	nonsynonymous SNV	p.P905L	rs201161628	4,07E-05	0,000349	0	0,01754386	0,465116279	3,61E-10	
S_6	FAM208A	3:56667953:-,C	frameshift insertion	p.Q956fs		0	0,001103	0	NA	0,318181818	NA	
S_6	NCL	2:232325429:T,A	nonsynonymous SNV	p.E254D	rs144444417	0	0	0	0	0,155737705	NA	
S_6	ZNF90	19:20229706:G,C	nonsynonymous SNV	p.S448T	rs368871975	0,000261	0	0	0	0,103825137	NA	
S_6	SLC6A13	12:330167:C,T	nonsynonymous SNV	p.A494T	rs61738753	0,000383	0	0	0	0,543478261	NA	
S_6	DENND4C	9:19334984:C,T	stopgain	p.R824X		8,13E-06	0	0	0	0,159090909	NA	
S_6	PTPRU	1:29638269:C,T	nonsynonymous SNV	p.R1021W		8,13E-06	0	0	0	0,356321839	NA	
S_6	ZNF431	19:21366002:G,A	nonsynonymous SNV	p.R299Q		8,13E-06	0	0	0	0,081081081	NA	
S_6	DYX1C1	15:55710265:T,A	nonsynonymous SNV	p.S372C		8,14E-06	0	0	0	0,166666667	NA	
S_6	ZNF431	19:21365885:T,A	nonsynonymous SNV	p.F260Y		8,14E-06	0	0	0	0,202797203	NA	
S_6	ZNF681	19:23926839:A,G	nonsynonymous SNV	p.S505P		8,14E-06	0	0	0	0,178082192	NA	

S_6	PRG4	1:186277168:C,A	nonsynonymous SNV	p.P639T	rs201504886	8,15E-06	0	0	0	0,086705202	NA
S_6	PIK3CA	3:178952085:A,G	nonsynonymous SNV	p.H1047R	rs121913279	8,17E-06	0	0	0,155844156	0,489795918	0,0030879
S_6	TJP1	15:30025352:C,T	nonsynonymous SNV	p.R561Q		8,17E-06	0	0	0	0,695652174	NA
S_6	ZNF729	19:22498764:A,G	nonsynonymous SNV	p.K849E		8,17E-06	0	0	0	0,159292035	NA
S_6	ZNF735	7:63680663:A,G	nonsynonymous SNV	p.K412E		8,17E-06	0	0	0	0,170212766	NA
S_6	ZNF99	19:22940859:T,C	nonsynonymous SNV	p.K618E	rs557629431	8,17E-06	0	0	0	0,181102362	NA
S_6	ZNF728	19:23158535:T,G	nonsynonymous SNV	p.Q535P		8,19E-06	0	0	0	0,134146341	NA
S_6	HRNR	1:152193339:C,T	nonsynonymous SNV	p.G256S		1,63E-05	0	0	0	0,07	NA
S_6	HRNR	1:152193371:C,G	nonsynonymous SNV	p.S245T	rs143710082	1,63E-05	0	0	0	0,066371681	NA
S_6	ZNF676	19:22363008:C,G	nonsynonymous SNV	p.R504P	rs202153135	1,63E-05	0	0	0	0,24	NA
S_6	ZNF761	19:53959137:G,T	nonsynonymous SNV	p.R459L		1,63E-05	0	0	0	0,264705882	NA
S_6	CRACR2B	11:831675:G,C	nonsynonymous SNV	p.R389T		1,94E-05	0	0	0	0,243902439	NA
S_6	ZNF480	19:52825625:T,A	nonsynonymous SNV	p.N331K		2,44E-05	0	0	0	0,115384615	NA
S_6	ZNF99	19:22941531:G,C	nonsynonymous SNV	p.Q394E	rs565513507	3,26E-05	0	0	0	0,1875	NA
S_6	ANKRD24	19:4219633:G,A	nonsynonymous SNV	p.E1017K		4,07E-05	0	0	0	0,217391304	NA
S_6	ZNF626	19:20808090:C,T	nonsynonymous SNV	p.G198E	rs202218510	4,07E-05	0	0	0	0,171717172	NA
S_6	IGFN1	1:201180581:G,A	nonsynonymous SNV	p.G2187E		4,58E-05	0	0	0,013333333	0,183284457	0,00045984
S_6	IGFN1	1:201180101:G,A	nonsynonymous SNV	p.R2027Q	rs527808016	4,91E-05	0	0	0	0,112947658	NA
S_6	TMPRSS9	19:2415773:G,A	nonsynonymous SNV	p.R526Q	rs201475068	8,95E-05	0	0	0	0,071428571	NA
S_6	LOC100129520	X:124456331:C,T	nonsynonymous SNV	p.T788M		0,000175	0	0	0	0,192307692	NA
S_6	AHNAK	11:62296271:G,A	nonsynonymous SNV	p.A1873V	rs145366859	0,000325	0	0	0,034482759	0,288	2,93E-05
S_6	TCHH	1:152082484:G,T	nonsynonymous SNV	p.T1070K	rs377407296	0,000604	0	0	0,054545455	0,385057471	2,38E-05
S_6	IGFN1	1:201179181:G,A	nonsynonymous SNV	p.M1720I	rs200673977	0,000777	0	0	0,020408163	0,279141104	2,81E-08
S_6	AGPAT1	6:32138783:C,A	nonsynonymous SNV	p.A89S		0	0	0	0	0,477272727	NA
S_6	ARHGEF18	19:7505352:C,T	nonsynonymous SNV	p.P176S		0	0	0	0	0,480874317	NA
S_6	ARID4A	14:58830921:C,-	frameshift deletion	p.A705fs		0	0	0	NA	0,7	NA
S_6	ATR	3:142168375:C,G	nonsynonymous SNV	p.G2611R		0	0	0	0	0,185185185	NA
S_6	BCAS4	20:49492555:C,T	nonsynonymous SNV	p.P171S		0	0	0	0	0,147435897	NA
S_6	BTN1A1	6:26509004:G,A	nonsynonymous SNV	p.A395T		0	0	0	0	0,109803922	NA
S_6	CDH1	16:68842406:G,A	stopgain	p.W156X		0	0	0	0,161290323	0,833333333	6,16E-06
S_6	CLEC4D	12:8671751:G,A	nonsynonymous SNV	p.E127K		0	0	0	0	0,13253012	NA
S_6	CLK2	1:155239368:C,T	nonsynonymous SNV	p.E104K		0	0	0	0	0,204301075	NA
S_6	CLSTN3	12:7295893:-,T	frameshift insertion	p.L611fs		0	0	0	NA	0,322580645	NA
S_6	CNNM2	10:104679621:G,A	nonsynonymous SNV	p.E462K		0	0	0	0	0,470588235	NA
S_6	COL6A3	2:238271913:C,T	nonsynonymous SNV	p.E1409K		0	0	0	0	0,139534884	NA
S_6	CSRNP3	2:166535550:-,A	frameshift insertion	p.G349fs		0	0	0	NA	0,385542169	NA
S_6	DIDO1	20:61510726:G,C	nonsynonymous SNV	p.S2194R		0	0	0	0	0,460606061	NA
S_6	DPCR1	6:30917854:C,T	nonsynonymous SNV	p.A538V		0	0	0	0,028846154	0,206993007	2,50E-05
S_6	DPCR1	6:30918254:G,C	nonsynonymous SNV	p.K671N		0	0	0	0,024096386	0,200723327	0,00029449
S_6	EDRF1	10:127436458:G,A	nonsynonymous SNV	p.M966I		0	0	0	0	0,515151515	NA
S_6	EHMT2	6:31857050:G,A	nonsynonymous SNV	p.R414W		0	0	0	0	0,112	NA
S_6	EPOR	19:11494880:C,T	nonsynonymous SNV	p.D2N		0	0	0	0	0,277777778	NA
S_6	FAM161B	14:74411377:C,G	nonsynonymous SNV	p.E259Q		0	0	0	0	0,140350877	NA
S_6	FBXO18	10:5966723:C,T	nonsynonymous SNV	p.S795F		0	0	0	0	0,307692308	NA
S_6	GCNT2	6:10529937:G,A	nonsynonymous SNV	p.A265T		0	0	0	0	0,087719298	NA
S_6	HDAC6	X:48681966:C,T	nonsynonymous SNV	p.L1053F		0	0	0	0	0,086956522	NA
S_6	HGC6.3	6:168377121:C,A	nonsynonymous SNV	p.G71V		0	0	0	0	0,181818182	NA
S_6	HMX1	4:8869772:C,A	stopgain	p.E232X		0	0	0	0	0,101851852	NA

S_6	IFT140	16:1636172:G,A	stopgain	p.Q372X		0	0	0	0	0,076086957	NA
S_6	IL12RB1	19:18182042:T,C	nonsynonymous SNV	p.T375A		0	0	0	0	0,283018868	NA
S_6	INSM1	20:20349846:C,G	nonsynonymous SNV	p.S312W		0	0	0	0	0,135135135	NA
S_6	KAT6B	10:76735849:-,CT	frameshift insertion	p.A585fs		0	0	0	NA	0,314984709	NA
S_6	KAT6B	10:76735852:-,C	frameshift insertion	p.H586fs		0	0	0	NA	0,311926606	NA
S_6	MAP4K4	2:102476146:G,C	nonsynonymous SNV	p.E508D		0	0	0	0	0,113207547	NA
S_6	MUC16	19:9066637:C,G	nonsynonymous SNV	p.E6937Q		0	0	0	0	0,222222222	NA
S_6	MUC5B	11:1276737:C,T	nonsynonymous SNV	p.R5339C		0	0	0	0	0,523076923	NA
S_6	MYCBP2	13:77631173:C,G	nonsynonymous SNV	p.R4462P		0	0	0	0	0,294117647	NA
S_6	MYH7	14:23901850:-,A	frameshift insertion	p.T167fs		0	0	0	NA	0,4375	NA
S_6	NAV3	12:78400573:G,A	nonsynonymous SNV	p.E419K		0	0	0	0	0,753846154	NA
S_6	NCL	2:232325426:A,T	nonsynonymous SNV	p.D255E	rs74931394	0	0	0	0,024390244	0,178988327	0,0044482
S_6	NDUFA11	19:5893211:A,G	nonsynonymous SNV	p.I135T		0	0	0	0	0,145833333	NA
S_6	NEB	2:152501064:T,A	nonsynonymous SNV	p.E2521V		0	0	0	0	0,21875	NA
S_6	NEIL3	4:178274579:G,A	nonsynonymous SNV	p.G386E		0	0	0	0	0,25	NA
S_6	NEO1	15:73585861:G,-	frameshift deletion	p.M1280fs		0	0	0	NA	0,337209302	NA
S_6	NKTR	3:42674119:ACTC,-	frameshift deletion	p.T193fs		0	0	0	NA	0,368421053	NA
S_6	NR1I2	3:119528912:G,T	nonsynonymous SNV	p.A68S		0	0	0	0	0,520408163	NA
S_6	NUP98	11:3724102:T,C	nonsynonymous SNV	p.K1035E		0	0	0	0	0,756097561	NA
S_6	PAF1	19:39877345:-,C	frameshift insertion	p.L351fs		0	0	0	NA	0,310679612	NA
S_6	PAF1	19:39877355:A,-	frameshift deletion	p.N347fs		0	0	0	NA	0,311111111	NA
S_6	PAF1	19:39877359:A,-	frameshift deletion	p.M346fs		0	0	0	NA	0,32183908	NA
S_6	PARVA	11:12399117:C,T	stopgain	p.Q15X		0	0	0	0	0,098039216	NA
S_6	PCLO	7:82452006:C,A	NA	NA		0	0	0	0,013157895	0,306666667	1,13E-05
S_6	PEX5L	3:179533774:C,T	nonsynonymous SNV	p.E261K		0	0	0	0	0,09375	NA
S_6	PHACTR1	6:13160441:GA,-	frameshift deletion	p.E141fs		0	0	0	NA	0,347826087	NA
S_6	PLA2G6	22:38531066:C,G	nonsynonymous SNV	p.D275H		0	0	0	0	0,087719298	NA
S_6	PLOD3	7:100859735:C,G	nonsynonymous SNV	p.E98D		0	0	0	0	0,24	NA
S_6	PRKD1	14:30396673:C,-	frameshift deletion	p.V16fs		0	0	0	NA	0,375	NA
S_6	PRODH2	19:36304123:T,C	nonsynonymous SNV	p.N21D		0	0	0	0	0,269230769	NA
S_6	QRICH2	17:74276533:C,T	NA	NA		0	0	0	0	0,608695652	NA
S_6	RPGR	X:38145295:-,T	frameshift insertion	p.G986fs		0	0	0	NA	0,316666667	NA
S_6	SERF2	15:44086123:C,-	frameshift deletion	p.P156fs		0	0	0	NA	0,310344828	NA
S_6	SERF2	15:44086138:G,-	frameshift deletion	p.A161fs		0	0	0	NA	0,404255319	NA
S_6	SHROOM4	X:50378430:C,T	nonsynonymous SNV	p.D215N		0	0	0	0	0,294117647	NA
S_6	SLC10A5	8:82606208:G,C	nonsynonymous SNV	p.L334V		0	0	0	0	0,105882353	NA
S_6	SLC25A29	14:100758676:C,T	nonsynonymous SNV	p.E220K		0	0	0	0	0,182481752	NA
S_6	SLC4A2	7:150768494:G,C	nonsynonymous SNV	p.E656Q		0	0	0	0	0,645714286	NA
S_6	SLC4A8	12:51873986:A,G	nonsynonymous SNV	p.M742V		0	0	0	0	0,058139535	NA
S_6	SMUG1	12:54577628:-,CA	frameshift insertion	p.E33fs		0	0	0	NA	0,394736842	NA
S_6	SMUG1	12:54577634:AG,-	frameshift deletion	p.S30fs		0	0	0	NA	0,394736842	NA
S_6	SPZ1	5:79616461:G,C	nonsynonymous SNV	p.E143Q		0	0	0	0	0,452830189	NA
S_6	TAF5	10:105139766:A,G	nonsynonymous SNV	p.N468S		0	0	0	0,12195122	0,789473684	3,66E-05
S_6	TBC1D15	12:72289754:C,T	stopgain	p.Q295X		0	0	0	0	0,144736842	NA
S_6	TCHH	1:152084159:C,G	nonsynonymous SNV	p.E512Q	rs547816345	0	0	0	0	0,09566787	NA
S_6	TEC	4:48147480:T,G	nonsynonymous SNV	p.I400L		0	0	0	0	0,256756757	NA
S_6	TMEM2	9:74345116:A,-	frameshift deletion	p.D546fs		0	0	0	NA	0,636363636	NA
S_6	TMEM79	1:156255100:C,T	nonsynonymous SNV	p.P28L		0	0	0	0	0,236363636	NA

S_6	TP53BP1	15:43748367:C,G	nonsynonymous SNV	p.K813N		0	0	0	0	0,078651685	NA	
S_6	TSPYL5	8:98288845:G,A	stopgain	p.Q410X		0	0	0	0	0,666666667	NA	
S_6	TTBK1	6:43251693:C,G	stopgain	p.S1072X		0	0	0	0	0,576923077	NA	
S_6	VN1R1	19:57967196:T,C	nonsynonymous SNV	p.H220R		0	0	0	0	0,541666667	NA	
S_6	ZC3H6	2:113057592:C,-	frameshift deletion	p.R67fs		0	0	0	NA	0,421052632	NA	
S_6	ZFHX3	16:72828742:A,-	frameshift deletion	p.T1699fs		0	0	0	NA	0,34375	NA	
S_6	ZNF117	7:64439050:C,G	nonsynonymous SNV	p.S300T		0	0	0	0	0,223529412	NA	
S_6	ZNF208	19:22153997:A,G	nonsynonymous SNV	p.L1280P		0	0	0	0	0,226666667	NA	
S_6	ZNF257	19:22271465:C,A	nonsynonymous SNV	p.Q305K		0	0	0	0	0,113207547	NA	
S_6	ZNF267	16:31927702:G,C	nonsynonymous SNV	p.S711T		0	0	0	0	0,275	NA	
S_6	ZNF292	6:87970761:-,A	frameshift insertion	p.E2472fs		0	0	0	NA	0,306451613	NA	
S_6	ZNF343	20:2472686:G,C	nonsynonymous SNV	p.L117V		0	0	0	0	0,062992126	NA	
S_6	ZNF430	19:21239907:C,G	nonsynonymous SNV	p.Q264E		0	0	0	0	0,144927536	NA	
S_6	ZNF480	19:52825623:A,G	nonsynonymous SNV	p.N331D		0	0	0	0	0,108695652	NA	
S_6	ZNF486	19:20308319:T,A	nonsynonymous SNV	p.I267N		0	0	0	0	0,170940171	NA	
S_6	ZNF491	19:11917747:G,T	nonsynonymous SNV	p.D327Y	rs555941733	0	0	0	0	0,253968254	NA	
S_6	ZNF502	3:44763369:A,G	nonsynonymous SNV	p.K354E		0	0	0	0	0,06185567	NA	
S_6	ZNF510	9:99521617:G,A	stopgain	p.Q499X		0	0	0	0,023809524	0,5	4,00E-05	
S_6	ZNF646	16:31092869:C,T	nonsynonymous SNV	p.R1742W		0	0	0	0	0,067114094	NA	
S_6	ZNF675	19:23836302:A,T	nonsynonymous SNV	p.I478K		0	0	0	0	0,164835165	NA	
S_6	ZNF680	7:63982003:A,G	nonsynonymous SNV	p.S377P		0	0	0	0	0,153846154	NA	
S_6	ZNF681	19:23926943:C,A	nonsynonymous SNV	p.R470I		0	0	0	0	0,107692308	NA	
S_6	ZNF695	1:247151011:C,T	nonsynonymous SNV	p.R269K		0	0	0	0	0,152542373	NA	
S_6	ZNF714	19:21300069:T,A	nonsynonymous SNV	p.F200Y		0	0	0	0	0,112781955	NA	
S_6	ZNF716	7:57528892:G,A	nonsynonymous SNV	p.R242K		0	0	0	0	0,208333333	NA	
S_6	ZNF716	7:57529309:C,A	nonsynonymous SNV	p.T381N		0	0	0	0	0,157894737	NA	
S_6	ZNF726	19:24115997:G,A	nonsynonymous SNV	p.R360K		0	0	0	0	0,124260355	NA	
S_6	ZNF728	19:23159456:C,T	nonsynonymous SNV	p.C228Y		0	0	0	0	0,19	NA	
S_6	ZNF729	19:22497090:A,C	nonsynonymous SNV	p.T291P		0	0	0	0	0,204918033	NA	
S_6	ZNF90	19:20229130:G,C	nonsynonymous SNV	p.R256P		0	0	0	0	0,186440678	NA	
S_6	ZNF91	19:23544157:T,C	nonsynonymous SNV	p.R510G		0	0	0	0	0,191011236	NA	
S_6	ZNF91	19:23544159:C,G	nonsynonymous SNV	p.S509T		0	0	0	0	0,195402299	NA	
S_6	ZNF99	19:22940355:T,C	nonsynonymous SNV	p.K786E		0	0	0	0	0,166666667	NA	
S_6	ZSCAN23	6:28403352:AAA,-	nonframeshift deletion	p.147_147del		0	0	0	NA	0,6	NA	
S_6	ZSWIM8	10:75553460:G,C	nonsynonymous SNV	p.E810Q		0	0	0	0	0,113043478	NA	
S_7	LAMA5	20:60906148:G,A	nonsynonymous SNV	p.P1197L	rs201679986	0,000139	0,000116	0	0	0,075085324	NA	
S_7	ABCB1	7:87148696:C,T	nonsynonymous SNV	p.R958Q	rs144369247	0,000252	0,000116	0	0	0,152173913	NA	
S_7	TCHH	1:152082095:G,A	nonsynonymous SNV	p.R1200W	rs199817873	0,000106	0,000238	0	0	0,175925926	NA	
S_7	PTBP3	9:114982644:GG,-	frameshift deletion	p.A484fs		0	0,001101	0	NA	0,34375	NA	
S_7	ZNF90	19:20229706:G,C	nonsynonymous SNV	p.S448T	rs368871975	0,000261	0	0	0	0,153846154	NA	
S_7	F5	1:169510399:G,C	nonsynonymous SNV	p.T1310R		8,13E-06	0	0	0	0,155635063	NA	not validated
S_7	ZNF600	19:53269940:T,G	nonsynonymous SNV	p.T357P		8,13E-06	0	0	0	0,098591549	NA	
S_7	ZNF431	19:21365885:T,A	nonsynonymous SNV	p.F260Y		8,14E-06	0	0	0	0,17	NA	
S_7	GTF3C1	16:27473680:C,T	nonsynonymous SNV	p.E1993K		8,16E-06	0	0	0	0,149122807	NA	
S_7	TUSC5	17:1183626:G,A	nonsynonymous SNV	p.A111T		8,17E-06	0	0	0	0,14	NA	
S_7	LRRC16A	6:25606460:G,A	nonsynonymous SNV	p.R1263Q		1,63E-05	0	0	0	0,111842105	NA	
S_7	CACNA1H	16:1265267:G,A	nonsynonymous SNV	p.V1683M		2,45E-05	0	0	0	0,0703125	NA	
S_7	ZNF679	7:63727074:A,G	nonsynonymous SNV	p.K355E		2,45E-05	0	0	0	0,153846154	NA	

S_7	SARDH	9:136599004:G,A	nonsynonymous SNV	p.R98W		4,97E-05	0	0	0	0,214285714	NA	
S_7	AHNAK	11:62290084:T,C	nonsynonymous SNV	p.I3935M		0	0	0	0	0,117647059	NA	
S_7	FMN1	15:33358875:G,T	nonsynonymous SNV	p.A404D		0	0	0	0	0,188405797	NA	
S_7	GAS2L3	12:101017881:G,T	nonsynonymous SNV	p.R433I		0	0	0	0	0,072463768	NA	
S_7	HTT	4:3076665:-,GCCGCCACC	nonframeshift insertion	p.Q38delinsQPPP		0	0	0	NA	0,533333333	NA	
S_7	IKBKB	8:42183519:C,T	nonsynonymous SNV	p.P671L		0	0	0	0	0,121495327	NA	
S_7	KIF19	17:72348360:T,A	nonsynonymous SNV	p.Y621N		0	0	0	0	0,096654275	NA	
S_7	LZTS3	20:3146241:G,A	nonsynonymous SNV	p.R363W		0	0	0	0	0,193548387	NA	
S_7	MAGEC1	X:140994252:C,A	nonsynonymous SNV	p.F354L	rs145693793	0	0	0	0	0,126436782	NA	
S_7	MUC4	3:195505838:G,C	nonsynonymous SNV	p.H4205D	rs59101491	0	0	0	0	0,078431373	NA	
S_7	NCOA3	20:46279815:GCAGCAGCA,-	nonframeshift deletion	p.1246_1249del		0	0	0	NA	0,358024691	NA	
S_7	OR2L2	1:248202206:A,G	nonsynonymous SNV	p.I213V		0	0	0	0	0,115942029	NA	
S_7	P2RY10	X:78216171:A,G	nonsynonymous SNV	p.N52D		0	0	0	0	0,179487179	NA	
S_7	SLC4A5	2:74458421:G,T	nonsynonymous SNV	p.T930N		0	0	0	0	0,183333333	NA	
S_7	TP53	17:7578380:CTGAGCAGCG,-	frameshift deletion	p.R49fs		0	0	0	NA	0,4	NA	
S_7	TRIO	5:14461334:C,T	nonsynonymous SNV	p.R1804C		0	0	0	0	0,069343066	NA	
S_7	WSB2	12:118481010:G,C	nonsynonymous SNV	p.P136A		0	0	0	0	0,082417582	NA	
S_7	ZNF253	19:20003219:A,G	nonsynonymous SNV	p.K388R		0	0	0	0	0,071428571	NA	
S_7	ZNF334	20:45130968:G,T	nonsynonymous SNV	p.A337D		0	0	0	0	0,194444444	NA	
S_7	ZNF431	19:21365861:G,T	nonsynonymous SNV	p.R252I		0	0	0	0	0,163043478	NA	
S_7	ZNF486	19:20308715:C,T	nonsynonymous SNV	p.T399I	rs562012086	0	0	0	0	0,118811881	NA	
S_7	ZNF534	19:52937217:T,A	nonsynonymous SNV	p.S9T		0	0	0	0	0,14893617	NA	
S_7	ZNF678	1:227843257:A,G	nonsynonymous SNV	p.K491E	rs201441176	0	0	0	0	0,12	NA	
S_7	ZNF716	7:57528987:C,T	nonsynonymous SNV	p.R274C		0	0	0	0	0,267605634	NA	
S_7	ZNF727	7:63538568:T,C	nonsynonymous SNV	p.S381P		0	0	0	0	0,052631579	NA	
S_7	ZNF729	19:22499899:A,G	nonsynonymous SNV	p.N1227S		0	0	0	0	0,163265306	NA	
S_7	ZNF845	19:53854904:T,C	nonsynonymous SNV	p.S326P		0	0	0	0	0,166666667	NA	
S_7	ZNF91	19:23544724:T,C	nonsynonymous SNV	p.K321E	rs201680274	0	0	0	0	0,136363636	NA	
S_8	TCHH	1:152082167:G,C	nonsynonymous SNV	p.L1176V	rs529242148	0	0	0,001	0	0,103174603	NA	
S_8	ZNF737	19:20728407:A,T	nonsynonymous SNV	p.F201Y	rs200285971	0,000106	0	0,005	0,024096386	0,286486486	2,22E-06	
S_8	ZNF737	19:20727675:C,A	nonsynonymous SNV	p.R445I	rs202023843	0,000367	0,000314	0	0	0,114068441	NA	
S_8	KIAA1462	10:30316448:C,T	nonsynonymous SNV	p.E877K	rs370847675	1,63E-05	0	0	0	0,1558753	NA	
S_8	ZNF90	19:20229706:G,C	nonsynonymous SNV	p.S448T	rs368871975	0,000261	0	0	0	0,193370166	NA	
S_8	TMPRSS6	22:37471180:G,C	nonsynonymous SNV	p.S446W	rs146063847	9,76E-05	0	0	0	0,162303665	NA	
S_8	F5	1:169510399:G,C	nonsynonymous SNV	p.T1310R		8,13E-06	0	0	0	0,094810379	NA	not validated
S_8	ZNF254	19:24310294:T,G	nonsynonymous SNV	p.S413A	rs554981870	8,13E-06	0	0	0	0,112565445	NA	
S_8	ZNF600	19:53269940:T,G	nonsynonymous SNV	p.T357P		8,13E-06	0	0	0	0,115384615	NA	
S_8	ZNF93	19:20044033:A,G	nonsynonymous SNV	p.N90S		8,13E-06	0	0	0	0,072289157	NA	
S_8	ZSCAN1	19:58564983:C,T	nonsynonymous SNV	p.S264F		8,13E-06	0	0	0	0,091317365	NA	
S_8	ZNF431	19:21365885:T,A	nonsynonymous SNV	p.F260Y		8,14E-06	0	0	0	0,192982456	NA	
S_8	ZNF681	19:23926839:A,G	nonsynonymous SNV	p.S505P		8,14E-06	0	0	0	0,194214876	NA	
S_8	ZNF729	19:22499464:A,G	nonsynonymous SNV	p.D1082G		8,15E-06	0	0	0	0,149659864	NA	
S_8	ZNF737	19:20728254:C,G	nonsynonymous SNV	p.S252T		8,15E-06	0	0	0,017094017	0,141342756	0,0012992	
S_8	ZNF727	7:63538736:C,T	nonsynonymous SNV	p.P437S		8,21E-06	0	0	0,0078125	0,118644068	0,0011352	
S_8	HS3ST6	16:1961976:G,A	nonsynonymous SNV	p.P215L		8,23E-06	0	0	0	0,189624329	NA	
S_8	ZNF850	19:37239768:T,C	nonsynonymous SNV	p.D693G		8,51E-06	0	0	0,010416667	0,138211382	0,00017179	
S_8	ZNF679	7:63727143:G,A	nonsynonymous SNV	p.E378K		1,63E-05	0	0	0	0,183246073	NA	
S_8	ZNF729	19:22499446:G,A	nonsynonymous SNV	p.C1076Y		1,63E-05	0	0	0	0,19047619	NA	

S_8	ZNF737	19:20727918:C,G	nonsynonymous SNV	p.S364T		1,63E-05	0	0	0,01	0,144366197	0,0008435
S_8	ZNF716	7:57529132:G,A	nonsynonymous SNV	p.R322K		2,45E-05	0	0	0	0,215625	NA
S_8	ZNF732	4:265139:C,T	nonsynonymous SNV	p.E502K		2,45E-05	0	0	0,02020202	0,209459459	0,00015372
S_8	MUC17	7:100683987:T,C	nonsynonymous SNV	p.I3097T	rs534619644	3,25E-05	0	0	0	0,139037433	NA
S_8	ZNF429	19:21720795:T,C	nonsynonymous SNV	p.V647A		3,27E-05	0	0	0	0,08423913	NA
S_8	MUC17	7:100682117:G,A	nonsynonymous SNV	p.G2474S	rs555953599	4,07E-05	0	0	0,033333333	0,187739464	1,30E-05
S_8	ZNF732	4:265103:T,C	nonsynonymous SNV	p.T514A		4,90E-05	0	0	0	0,083969466	NA
S_8	AHNAK	11:62296070:A,G	nonsynonymous SNV	p.V1940A		5,69E-05	0	0	0,002512563	0,08	2,13E-05
S_8	ZNF90	19:20215159:C,A	nonsynonymous SNV	p.H39N		8,13E-05	0	0	0	0,103448276	NA
S_8	IGFN1	1:201180436:G,A	nonsynonymous SNV	p.E2139K	rs375978523	9,19E-05	0	0	0,033613445	0,186111111	0,00026169
S_8	ZNF813	19:53994301:G,A	nonsynonymous SNV	p.R272K	rs559321027	0,000106	0	0	0,006410256	0,220512821	1,43E-09
S_8	TXNDC2	18:9887461:A,C	nonsynonymous SNV	p.I329L	rs202224858	0,00022	0	0	0,010135135	0,068376068	0,002911552
S_8	IGFN1	1:201180551:G,A	nonsynonymous SNV	p.G2177D	rs545602248	0,00211	0	0	0,021428571	0,243816254	5,25E-10
S_8	ACSL1	4:185691618:G,A	nonsynonymous SNV	p.P276S		0	0	0	0	0,277777778	NA
S_8	ADAMTS1	21:28212001:AT,-	frameshift deletion	p.E644fs		0	0	0	NA	0,454545455	NA
S_8	ADAMTS19	5:128864332:A,C	nonsynonymous SNV	p.E424D		0	0	0	0	0,142857143	NA
S_8	AHSG	3:186337689:G,A	nonsynonymous SNV	p.G240E		0	0	0	0	0,123076923	NA
S_8	ARHGAP23	17:36623293:T,A	nonsynonymous SNV	p.S457T		0	0	0	0	0,299065421	NA
S_8	ARHGAP23	17:36623294:C,T	nonsynonymous SNV	p.S457L		0	0	0	0	0,298611111	NA
S_8	ASIC3	7:150746227:C,G	nonsynonymous SNV	p.I85M		0	0	0	0	0,184210526	NA
S_8	ASNS	7:97498344:T,C	nonsynonymous SNV	p.N21S		0	0	0	0	0,157894737	NA
S_8	ATCAY	19:3907757:G,C	nonsynonymous SNV	p.K128N		0	0	0	0	0,170781893	NA
S_8	BMS1	10:43291948:A,C	nonsynonymous SNV	p.Q419P		0	0	0	0	0,146341463	NA
S_8	CCDC102A	16:57551978:A,G	NA	NA		0	0	0	0	0,172932331	NA
S_8	CCDC15	11:124857896:T,G	nonsynonymous SNV	p.Y592D		0	0	0	0,007843137	0,138755981	2,69E-07
S_8	CHD4	12:6707567:-,T	frameshift insertion	p.Q496fs		0	0	0	NA	0,317073171	NA
S_8	CHMP4A	14:24680763:C,-	frameshift deletion	p.E116fs		0	0	0	NA	0,302325581	NA
S_8	COL5A3	19:10085065:C,G	nonsynonymous SNV	p.G1121A		0	0	0	0	0,222222222	NA
S_8	CRAT	9:131857708:G,C	nonsynonymous SNV	p.L617V		0	0	0	0	0,132780083	NA
S_8	CYP2S1	19:41711869:G,A	nonsynonymous SNV	p.E391K		0	0	0	0	0,176470588	NA
S_8	CYP3A43	7:99436340:C,T	nonsynonymous SNV	p.T69M		0	0	0	0	0,113445378	NA
S_8	DIP2A	21:47976335:G,A	nonsynonymous SNV	p.R1109H		0	0	0	0	0,116766467	NA
S_8	DOCK10	2:225639747:T,G	nonsynonymous SNV	p.D1957A		0	0	0	0	0,136986301	NA
S_8	ENDOV	17:78403575:G,T	nonsynonymous SNV	p.S216I		0	0	0	0	0,233333333	NA
S_8	FAM172A	5:92956701:C,T	nonsynonymous SNV	p.E305K		0	0	0	0	0,262411348	NA
S_8	FAT4	4:126411809:C,T	nonsynonymous SNV	p.A4612V		0	0	0	0,014705882	0,191489362	3,98E-07
S_8	FGD1	X:54472657:G,A	nonsynonymous SNV	p.T924M		0	0	0	0	0,173529412	NA
S_8	FMN2	1:240371697:A,-	frameshift deletion	p.I1195fs		0	0	0	NA	0,357142857	NA
S_8	FMN2	1:240371699:CT,-	frameshift deletion	p.P1196fs		0	0	0	NA	0,357142857	NA
S_8	FSIP2	2:186664510:-,A	frameshift insertion	p.S3582fs		0	0	0	NA	0,315789474	NA
S_8	GC	4:72620804:C,T	nonsynonymous SNV	p.R352K		0	0	0	0	0,467889908	NA
S_8	GP6	19:55525520:A,G	nonsynonymous SNV	p.L598P		0	0	0	0	0,209677419	NA
S_8	HAS1	19:52217118:C,T	stopgain	p.W432X		0	0	0	0	0,148241206	NA
S_8	HIST1H2AD	6:26199286:C,G	nonsynonymous SNV	p.E62D		0	0	0	0	0,063829787	NA
S_8	IGFN1	1:201180199:G,A	nonsynonymous SNV	p.V2060I		0	0	0	0,01	0,155709343	0,00031654
S_8	IKBKG	X:153780388:G,T	nonsynonymous SNV	p.E125D		0	0	0	0	0,195652174	NA
S_8	KIAA2018	3:113378302:-,A	stopgain	p.S743_Q744delinsX		0	0	0	NA	0,317073171	NA
S_8	KMT2D	12:49425305:C,G	nonsynonymous SNV	p.V4395L		0	0	0	0	0,170454545	NA

S_8	LEPREL2	12:6947179:C,G	unknown	NA	0	0	0	0	0,24	NA
S_8	LOC93432	7:141920402:G,A	nonsynonymous SNV	p.G2031S	0	0	0	0,021164021	0,110294118	0,00347243
S_8	LOC93432	7:141920414:G,A	nonsynonymous SNV	p.G2035S	0	0	0	0,014492754	0,118811881	0,00010868
S_8	MAP3K12	12:53876906:G,A	nonsynonymous SNV	p.P561S	0	0	0	0	0,159090909	NA
S_8	MMP26	11:5013453:G,-	frameshift deletion	p.C256fs	0	0	0	NA	0,416666667	NA
S_8	MMP26	11:5013466:ACC,-	nonframeshift deletion	p.260_261del	0	0	0	NA	0,416666667	NA
S_8	MMRN1	4:90849008:A,G	nonsynonymous SNV	p.K340E	0	0	0	0	0,227272727	NA
S_8	MTPAP	10:30611403:G,C	nonsynonymous SNV	p.S379C	0	0	0	0	0,175	NA
S_8	MUC17	7:100680807:C,G	stopgain	p.S2037X	0	0	0	0	0,083333333	NA
S_8	MUC7	4:71347156:C,A	nonsynonymous SNV	p.P232Q	0	0	0	0,040816327	0,427350427	7,33E-07
S_8	NANOGNB	12:7917904:C,T	nonsynonymous SNV	p.T8M	0	0	0	0	0,061333333	NA
S_8	NAT10	11:34133658:T,G	nonsynonymous SNV	p.I15R	0	0	0	0	0,139037433	NA
S_8	NTN5	19:49174111:G,C	nonsynonymous SNV	p.Q45E	0	0	0	0	0,131498471	NA
S_8	NYAP1	7:100084566:C,A	nonsynonymous SNV	p.P64H	0	0	0	0	0,2	NA
S_8	OBSCN	1:228468323:G,A	nonsynonymous SNV	p.A2675T	0	0	0	0,008695652	0,23659306	8,06E-09
S_8	OR51T1	11:4903437:T,A	nonsynonymous SNV	p.M130K	0	0	0	0	0,191489362	NA
S_8	OSGIN2	8:90937131:T,G	nonsynonymous SNV	p.L341V	0	0	0	0	0,159090909	NA
S_8	OSMR	5:38904599:G,A	nonsynonymous SNV	p.E427K	0	0	0	0	0,227272727	NA
S_8	PCDHA10	5:140237798:C,T	nonsynonymous SNV	p.S722L	0	0	0	0	0,207100592	NA
S_8	PDS5A	4:39864961:T,G	nonsynonymous SNV	p.I921L	0	0	0	0	0,266666667	NA
S_8	PIKFYVE	2:209190097:G,C	nonsynonymous SNV	p.E854D	0	0	0	0	0,333333333	NA
S_8	RIF1	2:152320199:C,T	nonsynonymous SNV	p.P1389S	0	0	0	0	0,102941176	NA
S_8	RIN1	11:66102012:C,G	nonsynonymous SNV	p.E420Q	0	0	0	0	0,275167785	NA
S_8	SEC24C	10:75525555:CAG,-	NA	NA	0	0	0	NA	0,565217391	NA
S_8	SERF2	15:44086138:G,-	frameshift deletion	p.A161fs	0	0	0	NA	0,322033898	NA
S_8	SHARPIN	8:145154445:G,T	nonsynonymous SNV	p.P246T	0	0	0	0	0,06402439	NA
S_8	SLC25A14	X:129493050:T,G	nonsynonymous SNV	p.C205W	0	0	0	0	0,192307692	NA
S_8	SLC25A17	22:41173374:G,-	frameshift deletion	p.A79fs	0	0	0	NA	0,384615385	NA
S_8	TECTB	10:114046138:A,-	frameshift deletion	p.N158fs	0	0	0	NA	0,416666667	NA
S_8	TM9SF4	20:30729424:C,A	nonsynonymous SNV	p.S118R	0	0	0	0	0,126213592	NA
S_8	TRIM62	1:33631109:G,A	nonsynonymous SNV	p.A156V	0	0	0	0	0,168674699	NA
S_8	TTN	2:179402199:A,-	frameshift deletion	p.I24180fs	0	0	0	NA	0,315789474	NA
S_8	TTN	2:179545895:G,-	frameshift deletion	p.P9840fs	0	0	0	NA	0,6	NA
S_8	TXNDC2	18:9887284:C,G	nonsynonymous SNV	p.P270A	0	0	0	0,003322259	0,211382114	6,96E-17
S_8	TXNDC2	18:9887290:G,A	nonsynonymous SNV	p.A272T	0	0	0	0	0,226086957	NA
S_8	UGT2B7	4:69978360:T,C	nonsynonymous SNV	p.V499A	0	0	0	0	0,080808081	NA
S_8	XKR4	8:56436403:C,T	nonsynonymous SNV	p.P524S	0	0	0	0	0,184615385	NA
S_8	ZFHX3	16:72828742:A,-	frameshift deletion	p.T1699fs	0	0	0	NA	0,409090909	NA
S_8	ZNF100	19:21926850:T,C	nonsynonymous SNV	p.I97M	0	0	0	0	0,063545151	NA
S_8	ZNF138	7:64292444:A,G	nonsynonymous SNV	p.E128G	0	0	0	0	0,128440367	NA
S_8	ZNF254	19:24310427:A,G	nonsynonymous SNV	p.E457G	0	0	0	0,017857143	0,237851662	7,81E-08
S_8	ZNF254	19:24310450:A,G	nonsynonymous SNV	p.K465E	0	0	0	0,009708738	0,147826087	0,00033929
S_8	ZNF43	19:21990711:T,C	nonsynonymous SNV	p.K645E	0	0	0	0,010989011	0,148148148	0,0013485
S_8	ZNF43	19:21991466:A,T	nonsynonymous SNV	p.V393E	0	0	0	0	0,217228464	NA
S_8	ZNF430	19:21239907:C,G	nonsynonymous SNV	p.Q264E	0	0	0	0	0,223404255	NA
S_8	ZNF44	19:12384220:T,C	nonsynonymous SNV	p.K284E	0	0	0	0	0,081967213	NA
S_8	ZNF468	19:53344330:C,G	nonsynonymous SNV	p.S406T	0	0	0	0,006451613	0,171428571	5,35E-07
S_8	ZNF486	19:20308129:G,C	nonsynonymous SNV	p.D204H	0	0	0	0	0,233766234	NA

S_8	ZNF493	19:21606831:A,G	nonsynonymous SNV	p.E329G		0	0	0	0,006578947	0,146118721	1,30E-05
S_8	ZNF493	19:21607202:T,G	nonsynonymous SNV	p.S453A		0	0	0	0,01369863	0,184615385	0,001476
S_8	ZNF502	3:44763346:G,C	nonsynonymous SNV	p.S346T		0	0	0	0,0078125	0,337837838	7,01E-10
S_8	ZNF506	19:19917760:T,C	nonsynonymous SNV	p.I41V		0	0	0	0	0,100877193	NA
S_8	ZNF595	4:85978:G,A	unknown	NA		0	0	0	0,021276596	0,244680851	0,00011438
S_8	ZNF600	19:53268950:G,T	nonsynonymous SNV	p.L687I		0	0	0	0	0,178571429	NA
S_8	ZNF626	19:20829138:C,T	nonsynonymous SNV	p.R26Q		0	0	0	0	0,076923077	NA
S_8	ZNF676	19:22362758:A,T	nonsynonymous SNV	p.N587K	rs200753647	0	0	0	0	0,137931034	NA
S_8	ZNF676	19:22363189:C,T	nonsynonymous SNV	p.A444T		0	0	0	0,010526316	0,146892655	0,0008435
S_8	ZNF678	1:227842802:G,A	nonsynonymous SNV	p.R339K		0	0	0	0,020408163	0,233333333	5,19E-09
S_8	ZNF678	1:227843089:A,G	nonsynonymous SNV	p.K435E		0	0	0	0	0,1	NA
S_8	ZNF678	1:227843156:T,A	nonsynonymous SNV	p.V457E		0	0	0	0	0,134057971	NA
S_8	ZNF678	1:227843257:A,G	nonsynonymous SNV	p.K491E	rs201441176	0	0	0	0	0,124242424	NA
S_8	ZNF680	7:63982167:A,T	nonsynonymous SNV	p.F322Y	rs557997525	0	0	0	0	0,211009174	NA
S_8	ZNF714	19:21300069:T,A	nonsynonymous SNV	p.F200Y		0	0	0	0	0,141762452	NA
S_8	ZNF721	4:435773:C,T	nonsynonymous SNV	p.R828K		0	0	0	0	0,132450331	NA
S_8	ZNF726	19:24102293:C,T	nonsynonymous SNV	p.A41V		0	0	0	0	0,101796407	NA
S_8	ZNF726	19:24115997:G,A	nonsynonymous SNV	p.R360K		0	0	0	0	0,118589744	NA
S_8	ZNF727	7:63538656:A,G	nonsynonymous SNV	p.N410S		0	0	0	0,005586592	0,090634441	0,00063
S_8	ZNF729	19:22497090:A,C	nonsynonymous SNV	p.T291P		0	0	0	0	0,15625	NA
S_8	ZNF729	19:22498461:T,G	nonsynonymous SNV	p.S748A		0	0	0	0	0,147239264	NA
S_8	ZNF729	19:22498581:G,A	nonsynonymous SNV	p.V788I		0	0	0	0	0,152941176	NA
S_8	ZNF730	19:23329138:G,A	nonsynonymous SNV	p.R431K		0	0	0	0	0,213114754	NA
S_8	ZNF737	19:20728310:G,T	nonsynonymous SNV	p.D233E		0	0	0	0	0,106598985	NA
S_8	ZNF761	19:53958879:A,G	nonsynonymous SNV	p.H373R	rs543658141	0	0	0	0	0,138190955	NA
S_8	ZNF776	19:58265844:A,G	nonsynonymous SNV	p.Q449R	rs201940964	0	0	0	0,004016064	0,188356164	2,60E-13
S_8	ZNF816	19:53453178:G,C	nonsynonymous SNV	p.A617G		0	0	0	0	0,269230769	NA
S_8	ZNF829	19:37382466:G,T	nonsynonymous SNV	p.D409E		0	0	0	0	0,208333333	NA
S_8	ZNF844	19:12187275:G,C	nonsynonymous SNV	p.R447P		0	0	0	0,006993007	0,12371134	0,002911552
S_8	ZNF845	19:53854904:T,C	nonsynonymous SNV	p.S326P		0	0	0	0,016949153	0,246753247	4,72E-07
S_8	ZNF845	19:53855173:T,G	nonsynonymous SNV	p.D415E		0	0	0	0	0,080213904	NA
S_8	ZNF850	19:37240213:T,G	nonsynonymous SNV	p.I545L		0	0	0	0	0,152671756	NA
S_8	ZNF90	19:20229289:A,G	nonsynonymous SNV	p.E309G		0	0	0	0,01369863	0,147058824	5,13E-05
S_8	ZNF90	19:20229532:G,T	nonsynonymous SNV	p.S390I		0	0	0	0,014705882	0,181528662	2,43E-06
S_8	ZNF90	19:20229874:G,C	nonsynonymous SNV	p.S504T		0	0	0	0,006097561	0,111111111	0,0002444
S_8	ZNF91	19:23545076:A,C	nonsynonymous SNV	p.D203E		0	0	0	0,01459854	0,276315789	1,50E-09
S_8	ZNF93	19:20044524:A,G	nonsynonymous SNV	p.K254E	rs562648725	0	0	0	0,026315789	0,198237885	0,001972
S_8	ZNF93	19:20045112:A,G	nonsynonymous SNV	p.K450E		0	0	0	0,015503876	0,111111111	0,0053671
S_8	ZNF99	19:22941027:T,C	nonsynonymous SNV	p.K562E		0	0	0	0	0,124137931	NA
S_8	ZSCAN23	6:28403352:AAA,-	nonframeshift deletion	p.147_147del		0	0	0	NA	0,727272727	NA
S_9	ZNF267	16:31927690:G,A	nonsynonymous SNV	p.R707Q	rs146914846	0,00158	0,002093	0,002	NA	0,128205128	NA
S_9	ZNF709	19:12575478:G,A	nonsynonymous SNV	p.H420Y	rs200490873	4,07E-05	0,000116	0	NA	0,208333333	NA
S_9	ATCAY	19:3907734:G,A	nonsynonymous SNV	p.D121N	rs371914915	8,16E-05	0,000118	0	NA	0,412587413	NA
S_9	RPGR	X:38145198:T,-	frameshift deletion	p.E1018fs		0	0,009853	0	NA	0,47826087	NA
S_9	UBR4	1:19433211:C,T	nonsynonymous SNV	p.G4082D		8,14E-06	0	0	NA	0,172043011	NA
S_9	ZNF813	19:53995130:T,G	nonsynonymous SNV	p.D548E		8,14E-06	0	0	NA	0,294117647	NA
S_9	PPFIBP1	12:27841969:G,A	nonsynonymous SNV	p.V693I	rs539499516	1,63E-05	0	0	NA	0,565217391	NA
S_9	ZFAT	8:135524750:G,A	nonsynonymous SNV	p.A1048V		1,63E-05	0	0	NA	0,171875	NA

S_9	CACNG5	17:64873629:G,A	nonsynonymous SNV	p.R60Q		4,07E-05	0	0	NA	0,480582524	NA
S_9	ZNF626	19:20808090:C,T	nonsynonymous SNV	p.G198E	rs202218510	4,07E-05	0	0	NA	0,225	NA
S_9	IGFN1	1:201180581:G,A	nonsynonymous SNV	p.G2187E		4,58E-05	0	0	NA	0,064516129	NA
S_9	ZNF626	19:20807669:G,C	nonsynonymous SNV	p.D338E		4,89E-05	0	0	NA	0,073529412	NA
S_9	ZNF737	19:20727799:C,T	nonsynonymous SNV	p.E404K		8,14E-05	0	0	NA	0,134328358	NA
S_9	IGFN1	1:201180436:G,A	nonsynonymous SNV	p.E2139K	rs375978523	9,19E-05	0	0	NA	0,055248619	NA
S_9	TMEM249	8:145577021:C,A	nonsynonymous SNV	p.Q200H		0,00131	0	0	NA	0,173333333	NA
S_9	IGFN1	1:201180551:G,A	nonsynonymous SNV	p.G2177D	rs545602248	0,00211	0	0	NA	0,133333333	NA
S_9	MARCH	17:60814271:C,A	nonsynonymous SNV	p.G320W		0	0	0	NA	0,160493827	NA
S_9	AKR1B1	7:134132049:C,A	NA	NA		0	0	0	NA	0,435897436	NA
S_9	ARID4A	14:58817859:C,-	frameshift deletion	p.D491fs		0	0	0	NA	0,342857143	NA
S_9	ATM	11:108186817:A,G	nonsynonymous SNV	p.T2059A		0	0	0	NA	0,814814815	NA
S_9	AXIN2	17:63533766:C,T	nonsynonymous SNV	p.R463H		0	0	0	NA	0,07804878	NA
S_9	CDK18	1:205498502:C,T	nonsynonymous SNV	p.R375C		0	0	0	NA	0,263736264	NA
S_9	CHPF	2:220406741:C,G	nonsynonymous SNV	p.G162A		0	0	0	NA	0,433121019	NA
S_9	COQ4	9:131085413:C,A	nonsynonymous SNV	p.N63K		0	0	0	NA	0,1125	NA
S_9	CTCFL	20:56075415:T,G	nonsynonymous SNV	p.H369P		0	0	0	NA	0,111111111	NA
S_9	EGR2	10:64574021:G,A	nonsynonymous SNV	p.S126F		0	0	0	NA	0,487804878	NA
S_9	EPG5	18:43450653:C,T	nonsynonymous SNV	p.C2035Y		0	0	0	NA	0,536231884	NA
S_9	ERBB3	12:56488340:G,T	nonsynonymous SNV	p.G620V		0	0	0	NA	0,277777778	NA
S_9	EVI5L	19:7926901:G,T	nonsynonymous SNV	p.W547C		0	0	0	NA	0,489583333	NA
S_9	GFOD1	6:13365145:C,A	nonsynonymous SNV	p.A232S		0	0	0	NA	0,407960199	NA
S_9	KIAA1210	X:118223663:C,G	nonsynonymous SNV	p.K510N		0	0	0	NA	0,761904762	NA
S_9	KRT7	12:52627318:G,A	nonsynonymous SNV	p.A80T		0	0	0	NA	0,175	NA
S_9	KRTAP4-5	17:39305774:-,TGGCAGCAGCTGGGG	nonframeshift insertion	p.Q82delinsHPSCCQ		0	0	0	NA	0,488372093	NA
S_9	LOC100996634	6:109591553:T,-	frameshift deletion	p.P586fs		0	0	0	NA	0,322580645	NA
S_9	LRRC16B	14:24526163:C,A	nonsynonymous SNV	p.A331E		0	0	0	NA	0,41025641	NA
S_9	MAGED2	X:54837736:C,G	nonsynonymous SNV	p.I300M		0	0	0	NA	0,428571429	NA
S_9	MOXD1	6:132649643:C,A	stopgain	p.E252X		0	0	0	NA	0,5	NA
S_9	OGFOD3	17:80373349:G,A	nonsynonymous SNV	p.R77C		0	0	0	NA	0,178137652	NA
S_9	PARP15	3:122338675:G,T	stopgain	p.E116X		0	0	0	NA	0,259259259	NA
S_9	PLVAP	19:17476247:G,T	nonsynonymous SNV	p.Q343K		0	0	0	NA	0,098712446	NA
S_9	PTOV1	19:50360340:A,G	nonsynonymous SNV	p.S223G		0	0	0	NA	0,278350515	NA
S_9	RIN2	20:19970801:G,T	nonsynonymous SNV	p.E638D		0	0	0	NA	0,171974522	NA
S_9	RPTOR	17:78858915:G,T	nonsynonymous SNV	p.Q650H		0	0	0	NA	0,442105263	NA
S_9	SOX11	2:5833592:G,A	nonsynonymous SNV	p.D247N		0	0	0	NA	0,167630058	NA
S_9	TMEM132C	12:129189813:C,T	nonsynonymous SNV	p.P767L		0	0	0	NA	0,497816594	NA
S_9	TMPRSS13	11:117789342:T,C	nonsynonymous SNV	p.Q78R	rs75037497	0	0	0	NA	0,060240964	NA
S_9	TRPV2	17:16329481:G,A	stopgain	p.W331X		0	0	0	NA	0,828571429	NA
S_9	ZNF254	19:24309932:G,C	nonsynonymous SNV	p.R292P		0	0	0	NA	0,113636364	NA
S_9	ZNF680	7:63981835:T,G	nonsynonymous SNV	p.T433P		0	0	0	NA	0,138888889	NA
S_9	ZNF680	7:63981836:A,T	nonsynonymous SNV	p.N432K		0	0	0	NA	0,138888889	NA
S_9	ZNF99	19:22940607:T,C	nonsynonymous SNV	p.K702E		0	0	0	NA	0,125	NA
S_9	ZSCAN4	19:58187686:C,G	nonsynonymous SNV	p.A58G		0	0	0	NA	0,5	NA

Supplementary Table S3. Identification of somatic mutations in brain metastases.

Patient ID	Gene Symbol	Genomic Position	Function	Protein Change	dbSNP142	MAF in ExAC	MAF in ESP eur	MAF in 1KG eur	VAF
22549	C3	19:6682224:C,T	nonsynonymous SNV	p.D1397N		0	0	0	0,07123288
22549	C3	19:6697467:G,A	nonsynonymous SNV	p.S895F		0	0	0	0,15618661
22549	FGA	4:155505403:C,T	nonsynonymous SNV	p.G825D		0	0	0	0,26086957
22549	SERPIND1	22:21133797:C,T	nonsynonymous SNV	p.T66I		1,63E-05	0	0	0,42857143
22549	TFPI	2:188348889:G,A	nonsynonymous SNV	p.S197F		0	0	0	0,33018868
22549	VWF	12:6173441:C,T	nonsynonymous SNV	p.G468D		0	0	0	0,20731707
22624	SERPINI1	3:167525045:C,T	stopgain	p.Q299X		0	0	0	0,07142857
25318	SERPINI1	3:167508172:C,T	nonsynonymous SNV	p.S88F		0	0	0	0,07142857
26165	SERPINA10	14:94752464:C,T	nonsynonymous SNV	p.R375K		0	0	0	0,0516129
26974	SERPINA5	14:95053949:G,A	nonsynonymous SNV	p.G84R		0	0	0	0,056
27596	C3	19:6712610:C,T	nonsynonymous SNV	p.R343H	rs373511900	1,63E-05	0	0	0,07272727
27596	C3	19:6714417:G,A	nonsynonymous SNV	p.S182F		0	0	0	0,0625
27596	F5	1:169511750:G,T	nonsynonymous SNV	p.Q860K		0	0	0	0,07368421
27596	PLAT	8:42036515:G,A	nonsynonymous SNV	p.T431I		0	0	0	0,24
27596	SERPINI1	3:167507109:C,T	stopgain	p.Q65X		0	0	0	0,10638298
27599	BDKRB1	14:96730683:A,T	nonsynonymous SNV	p.N222Y		0	0	0	0,16666667
27599	F12	5:176832324:C,T	nonsynonymous SNV	p.E133K		0	0	0	0,07142857
27599	F7	13:113761189:C,T	nonsynonymous SNV	p.T32I		0	0	0	0,3125
27599	THBD	20:23029487:C,A	nonsynonymous SNV	p.G219C		0	0	0	0,13888889
27602	THBD	20:23028755:C,T	nonsynonymous SNV	p.E463K		0	0	0	0,0647482
27699	F2	11:46742361:C,T	nonsynonymous SNV	p.P96L		0	0	0	0,35051546
27699	F5	1:169525883:C,T	NA	NA		0	0	0	0,11904762
27699	F5	1:169525950:C,T	nonsynonymous SNV	p.A296T		2,44E-05	0	0	0,11904762
27743	SERPINE2	2:224842275:C,T	nonsynonymous SNV	p.R381Q		2,44E-05	0	0	0,05376344
28566	C5	9:123776279:G,A	nonsynonymous SNV	p.T710I		0	0	0	0,05583756
28566	C5	9:123792717:C,T	nonsynonymous SNV	p.G239D		0	0	0	0,075
28566	CD46	1:207940459:G,A	nonsynonymous SNV	p.G259S		0	0	0	0,05376344
28566	CD46	1:207940957:C,T	nonsynonymous SNV	p.P288S		0	0	0	0,08256881
28566	F10	13:113803787:A,C	nonsynonymous SNV	p.K475Q		0	0	0	0,05940594
28566	F7	13:113760165:C,T	stopgain	p.Q4X		0	0	0	0,06666667
28566	PLAT	8:42039493:C,T	nonsynonymous SNV	p.R238H		0	0	0	0,10204082
28566	SERPINB2	18:61570529:C,A	nonsynonymous SNV	p.S413Y		0	0	0	0,22222222

28566	SERPIND1	22:21133826:G,A	nonsynonymous SNV	p.D76N		0	0	0	0,23076923
28566	SERPINI1	3:167507097:G,C	nonsynonymous SNV	p.E61Q		0	0	0	0,05940594
28566	VWF	12:6172191:C,T	nonsynonymous SNV	p.A488T		0	0	0	0,07142857
30411	SERPINI1	3:167508172:C,T	nonsynonymous SNV	p.S88F		0	0	0	0,1
31337	C3	19:6712584:G,T	nonsynonymous SNV	p.P352T		0	0	0	0,08139535
31337	C5	9:123753546:C,T	nonsynonymous SNV	p.R955K		0	0	0	0,05128205
31337	F5	1:169511621:C,T	nonsynonymous SNV	p.G903R		4,07E-05	0	0	0,29411765
31337	KLKB1	4:187155200:C,T	nonsynonymous SNV	p.H106Y		0	0	0	0,05755396
31337	SERPINC1	1:173884040:G,A	nonsynonymous SNV	p.S20F	rs369828221	8,13E-06	0	0	0,08955224
31337	VWF	12:6167090:C,T	nonsynonymous SNV	p.A552T		8,13E-06	0	0	0,05319149
H09-25788	C5	9:123778538:C,A	stopgain	p.E664X		0	0	0	0,30674003
H09-25788	F5	1:169510328:A,G	nonsynonymous SNV	p.F1334L	rs147741798	4,07E-05	0	0	0,08333333
H09-25788	SERPINA10	14:94754858:C,T	nonsynonymous SNV	p.V253I		0	0	0	0,12195122
H13-753	C3	19:6692955:C,T	nonsynonymous SNV	p.V1124M		0	0	0	0,06282723
H13-753	C3	19:6709765:C,T	nonsynonymous SNV	p.R592Q	rs121909583	0	0	0	0,05208333
H13-753	C3	19:6719233:C,T	nonsynonymous SNV	p.V86I		0	0	0	0,08823529
H13-753	PLAU	10:75674590:C,T	stopgain	p.Q279X		8,13E-06	0	0	0,07608696
H13-753	PLG	6:161139838:C,T	nonsynonymous SNV	p.S355F		0	0	0	0,14285714
H13-753	SERPINF2	17:1657421:C,T	stopgain	p.Q293X		0	0	0	0,12
H13-753	VWF	12:6181527:C,T	nonsynonymous SNV	p.G360D		0	0	0	0,0610687
S_3	C1QB	1:22986016:G,A	nonsynonymous SNV	p.D23N		0,00013	0	0	0,52366566
S_10	C5	9:123739039:T,G	nonsynonymous SNV	p.N1268T		0	0	0	0,240625
S_10	FGA	4:155507877:C,T	nonsynonymous SNV	p.G235E		0	0	0	0,2231405
S_10	SERPINA1	14:94848980:C,T	nonsynonymous SNV	p.E199K		0	0	0	0,09027778
22357	*NO MUTATIONS*	-	-	-	-	-	-	-	-
24957	*NO MUTATIONS*	-	-	-	-	-	-	-	-
25299	*NO MUTATIONS*	-	-	-	-	-	-	-	-
25398	*NO MUTATIONS*	-	-	-	-	-	-	-	-
26208	*NO MUTATIONS*	-	-	-	-	-	-	-	-
26266	*NO MUTATIONS*	-	-	-	-	-	-	-	-
31196	*NO MUTATIONS*	-	-	-	-	-	-	-	-
32187	*NO MUTATIONS*	-	-	-	-	-	-	-	-
S_6	*NO MUTATIONS*	-	-	-	-	-	-	-	-
S_7	*NO MUTATIONS*	-	-	-	-	-	-	-	-
S_8	*NO MUTATIONS*	-	-	-	-	-	-	-	-
S_11	*NO MUTATIONS*	-	-	-	-	-	-	-	-

Supplementary Table S4. Gene enrichment analysis performed by gene of interested.

Term	Overlap	P-value	Z-score	C-score	Genes
Acute myeloid leukemia_Homo sapiens_hsa05221	7/57	0.5867	-2.0416	6.20562E-11	IKBKB; PIK3CA; FLT3; ARAF; BRAF; IKBKG; PML
Thyroid cancer_Homo sapiens_hsa05216	4/29	0.6368	-1.7863	5.42934E-11	CDH1; TPR; BRAF; TP53
Complement and coagulation cascades_Homo sapiens_hsa04610	7/79	0.7591	-1.6285	4.94981E-11	C1QB; FGA; C5; SERPINA1; VWF; CD46; F5
Nicotine addiction_Homo sapiens_hsa05033	2/40	0.7528	-1.6247	4.9384E-11	CACNA1A; GRIN2C
Bladder cancer_Homo sapiens_hsa05219	5/41	0.7613	-1.5595	4.74025E-11	DAPK1; CDH1; ARAF; BRAF; TP53
Galactose metabolism_Homo sapiens_hsa00052	3/30	0.6493	-1.5577	4.73461E-11	AKR1B1; GCK; HK1
Fructose and mannose metabolism_Homo sapiens_hsa00051	4/32	0.6730	-1.4868	4.51911E-11	PFKFB3; AKR1B1; ALDOC; HK1
Type II diabetes mellitus_Homo sapiens_hsa04930	5/48	0.8131	-1.4702	4.46868E-11	IKBKB; PIK3CA; CACNA1A; GCK; HK1
ECM-receptor interaction_Homo sapiens_hsa04512	8/82	0.7770	-1.4695	4.46647E-11	LAMA5; VWF; COL4A2; COL6A2; COL6A3; COL6A6; GP6; THBS3
Base excision repair_Homo sapiens_hsa03410	4/33	0.6842	-1.4344	4.35973E-11	SMUG1; NEIL3; LIG1; MUTYH

Richichi C. et al. Supplementary Table S5

Supplementary Table S5. Mutations' frequency in the coagulation and complement pathway listed by gene between primary tumors from cBioPortal (TCGA, in blue) database and brain metastases of our cohort (in red).

Gene	breast_TCGA_Primary	lung_TCGA_Primary	kidney_TCGA_Primary	breast_Metastasis	lung_Metastasis	kidney_Metastasis	total_TCGA_Primary	total_Metastasis	breast_pvalue	lung_pvalue	kidney_pvalue	total_pvalue
BDKRB1	5	10	1	0	1	0	16	1	1	0,4349334	1	0,245937
C1QB	1	11	0	1	0	0	12	1	0,0788205	1	1	0,21846
C3	13	25	22	2	1	1	60	4	0,068332	0,659439	0,750052286	0,0209385
C5	6	11	7	2	1	1	24	4	0,04356	0,4349334	0,351384	1,22E-03
CD46	4	5	1	0	1	0	10	1	1	0,353533714	1	0,206756
CFH	18	40	5	0	0	0	63	0	1	1	1	1
F10	4	6	1	0	1	0	11	1	1	0,353533714	1	0,213378
F11	3	14	2	0	0	0	19	0	1	1	1	1
F12	4	6	9	0	1	0	19	1	1	0,353533714	1	0,246171391
F2	3	16	5	0	1	0	24	1	1	0,5457705	1	0,269452615
F3	1	5	0	0	0	0	6	0	1	1	1	1
F5	21	72	16	1	2	1	109	4	0,4036428	0,588775846	0,646767	0,0679272
F7	2	11	1	0	2	0	14	2	1	0,0754875	1	0,043428
FGA	10	29	4	2	0	0	43	2	0,058938	1	1	0,1847945
KLKB1	5	16	2	0	0	1	23	1	1	1	0,1292775	0,269452615
PLAT	6	12	4	0	2	0	22	2	1	0,0754875	1	0,0679272
PLAU	4	6	5	1	0	0	15	1	0,113994	1	1	0,245937
PLG	9	31	6	1	0	0	46	1	0,195463714	1	1	0,449841333
PROC	3	4	4	0	0	0	11	0	1	1	1	1
SERPINA1	4	8	9	1	0	0	21	1	0,113994	1	1	0,2597925
SERPINA10	2	9	2	1	0	1	13	2	0,090787714	1	0,1292775	0,043428
SERPINA5	5	13	0	1	0	0	18	1	0,129151	1	1	0,245937
SERPINB2	1	17	0	0	1	0	18	1	1	0,5457705	1	0,245937
SERPINC1	6	12	0	0	0	1	18	1	1	1	0	0,245937
SERPIND1	2	7	10	1	1	0	19	2	0,090787714	0,35708475	1	0,06361575
SERPINE1	5	12	0	0	0	0	17	0	1	1	1	1
SERPINE2	2	7	1	0	0	1	10	1	1	1	0,1292775	0,206756
SERPINF2	4	4	2	1	0	0	10	1	0,113994	1	1	0,206756
SERPING1	3	13	3	0	0	0	19	0	1	1	1	1
SERPINI1	6	11	3	1	4	0	20	5	0,141463385	3,30E-04	1	0
TFPI	3	3	1	1	0	0	7	1	0,113994	1	1	0,1847945
THBD	3	5	0	0	2	0	8	2	1	0,024354	1	0,0209385
VWF	19	54	27	2	1	1	100	4	0,0863148	1	0,78631575	0,06361575
**Total Number of Patients**	**1258**	**783**	**904**	**12**	**10**	**7**	**2945**	**29**				
